# Droplet digital PCR-based analyses for robust, rapid, and sensitive molecular diagnostics of gliomas

**DOI:** 10.1186/s40478-022-01335-6

**Published:** 2022-03-31

**Authors:** Marietta Wolter, Jörg Felsberg, Bastian Malzkorn, Kerstin Kaulich, Guido Reifenberger

**Affiliations:** 1 Institute of Neuropathology, Medical Faculty, Heinrich Heine University, and University Hospital Düsseldorf, Moorenstraße 5, 40225 Düsseldorf, Germany; 2 German Cancer Consortium (DKTK), German Cancer Research Center (DKFZ) Heidelberg, Partner Site Essen, Düsseldorf, Germany

**Keywords:** DNA copy number variation, Droplet digital PCR, Glioma, Molecular diagnostics, Mutation, Single nucleotide variation

## Abstract

Classification of gliomas involves the combination of histological features with molecular biomarkers to establish an integrated histomolecular diagnosis. Here, we report on the application and validation of a set of molecular assays for glioma diagnostics based on digital PCR technology using the QX200™ Droplet Digital™ PCR (ddPCR) system. The investigated ddPCR-based assays enable the detection of diagnostically relevant glioma-associated mutations in the *IDH1, IDH2, H3-3A, BRAF*, and *PRKCA* genes, as well as in the *TERT* promoter. In addition, ddPCR-based assays assessing diagnostically relevant copy number alterations were studied, including 1p/19q codeletion, gain of chromosome 7 and loss of chromosome 10 (+ 7/-10), *EGFR* amplification, duplication of the *BRAF* locus, and *CDKN2A* homozygous deletion. Results obtained by ddPCR were validated by other methods, including immunohistochemistry, Sanger sequencing, pyrosequencing, microsatellite analyses for loss of heterozygosity, as well as real-time PCR- or microarray-based copy number assays. Particular strengths of the ddPCR approach are (1) its high analytical sensitivity allowing for reliable detection of mutations even with low mutant allele frequencies, (2) its quantitative determination of mutant allele frequencies and copy number changes, and (3) its rapid generation of results within a single day. Thus, in line with other recent studies our findings support ddPCR analysis as a valuable approach for molecular glioma diagnostics in a fast, quantitative and highly sensitive manner.

**Supplementary Information:**

The online version contains supplementary material available at 10.1186/s40478-022-01335-6.

## Introduction

Gliomas comprise a heterogeneous group of primary central nervous system (CNS) tumors that are classified into distinct tumor types by the integration of histological features and specific molecular biomarkers according to the recent World Health Organization (WHO) classification of CNS tumours [1] that considers the recommendations by the Consortium to Inform Molecular and Practical Approaches to CNS Tumour Taxonomy—Not Officially WHO (cIMPACT-NOW) [2, 3]. Accordingly, diffuse astrocytic and oligodendroglial gliomas are separated into distinct tumor types by histology and one or more of the following molecular biomarkers [1, 2]: (i) Missense mutations in codon 132 of the isocitrate dehydrogenase 1 gene (*IDH1*) or codon 172 of the *IDH2* gene (“IDH mutation”), (ii) codeletion of whole chromosome arms 1p and 19q (“1p/19q codeletion”), (iii) mutations in the alpha thalassemia/mental retardation syndrome X-linked (*ATRX*) gene and/or loss of nuclear ATRX expression, (iv) p.K28M missense mutation in the histone 3 genes *H3-3A (H3F3A)*, *H3C2* (*HIST1H3B)* or *H3C3* (*HIST1H3C)* affecting amino acid 27 in the mature H3.3 or H3.1 proteins (H3 K27M) [4], (v) p.G35R or p.G35V missense mutations in *H3-3A* affecting amino acid 34 in the mature H3.3 protein (H3 G34R/V) [4], (vi) hotspot mutations in the telomerase reverse transcriptase (*TERT)* gene promoter, (vii) epidermal growth factor receptor (*EGFR)* gene amplification, (viii) whole chromosome 7 gain combined with whole chromosome 10 loss (+ 7/–10 signature), and (ix) homozygous deletions on 9p21 involving the *CDKN2A* and *CDKN2B* gene loci (“*CDKN2A/CDKN2B* homozygous deletion”) [2, 5]. Additional molecular biomarkers are important for the diagnostics of gliomas with circumscribed growth, such as pilocytic astrocytoma, pleomorphic xanthoastrocytoma and chordoid glioma [1]. These include among others (i) *BRAF* gene duplications associated with *KIAA1549*-*BRAF* gene fusions [6–8], (ii) missense mutations affecting codon 600 of the *BRAF* gene, in particular BRAF V600E [9], and (iii) a missense mutation affecting codon 463 of the *PRKCA* gene (PRKCA D463H) [10, 11].

Certain glioma-associated biomarkers can be assessed by immunohistochemistry, including IDH1 R132H [12], H3 K27M and H3 G34R/V [13–15], loss of nuclear ATRX expression [16], and BRAF V600E mutation [17]. Detection of other biomarkers requires cytogenetic or molecular genetic analyses [1]. Recently, high-throughput molecular approaches involving gene panel next generation sequencing (NGS) [18–20] and DNA methylome profiling [21] have gained importance in glioma diagnostics. Each technique has specific advantages and limitations, e.g. relatively low analytical sensitivity of mutation detection by Sanger sequencing [22], false-positive results by fluorescence in situ hybridization (FISH) analysis for 1p/19q codeletion [23], as well as higher costs and prolonged analysis time of high-throughput approaches [24].

Droplet digital PCR (ddPCR) allows for the rapid and sensitive detection of DNA sequence and copy number variations [25, 26]. In principle, a large number (up to 20,000) of small partitions ("droplets") are generated before the PCR reaction is performed [25]. PCR products are then detected by fluorescence analysis of each individual droplet, with positive and negative droplets being counted and analysed by Poisson statistics [25]. Thereby, ddPCR allows for quantitative assessments without the need of traditional standards, as well as highly sensitive detection of rare sequence variants in an abundant wildtype background [25, 26].

Here, we report on the successful application and comprehensive validation of a set of previously reported and newly established ddPCR-based assays for rapid, sensitive and quantitative detection of eleven diagnostically relevant glioma-associated molecular biomarkers. Our data further support ddPCR analysis as a valuable tool to facilitate and speed up the routine diagnostic assessment of gliomas.

## Materials and methods

### Patient samples

Formalin-fixed and paraffin-embedded (FFPE) or fresh-frozen tumor tissue samples from patients with different types of gliomas were retrieved from the CNS tumor tissue bank or the institutional archive at the Department of Neuropathology, Heinrich Heine University, Düsseldorf, Germany. All tumors were re-classified according to the WHO classification of CNS tumors 2021 [1]. In total, 248 tumor samples were studied. Table 1 provides an overview of the various glioma types evaluated by the different ddPCR assays. As constitutional controls, we investigated 14 leukocyte DNA samples extracted from peripheral blood. Patients gave their written informed consent for the storage of their tissue and blood samples in the CNS tumor tissue bank and the use of these samples for research purposes as approved by the Institutional Review Board of the Medical Faculty, Heinrich Heine University Düsseldorf (study number: 3005). The use of archival tissue samples for research purposes was also approved by the Institutional Review Board (study number: 3562). The current study was additionally approved by a project-specific ethics vote (study number: 2019–702).

**Table 1:** Summary of the individual samples investigated by one or more ddPCR-based assays and appropriate other methods for validation

	Number of cases	TERTp mutation	IDH mutation	BRAF mutation	H3-3A mutation	PRKCA mutation	1p/19q co-deletion*	deletion on chr10*	gain on chr7	EGFR amplification	EGFRvIII deletion	BRAF duplication	CDKN2A deletion
Astrocytoma, IDH-mutant, CNS WHO grade 2	12	7 (1/6)	6 (6/0)	1 (0/1)	–	–	–	1 (1/0/0)	–	–	–	–	6 (1/5)
Astrocytoma, IDH-mutant, CNS WHO grade 3	7	3 (0/3)	2 (2/0)	–	–	–	1 (0/1/0)	–	–	–	–	–	3 (1/2)
Astrocytoma, IDH-mutant, CNS WHO grade 4	11	3 (0/3)	5 (5/0)	–	1 (0/1)	–	–	1 (1/0/0)	1 (0/1)	–	–	–	5 (4/1)
Glioblastoma, IDH-wildtype, CNS WHO grade 4	122	36 (36/0)	9 (0/9)	12 (2/10)	1 (0/1)	5 (0/5)	4 (0/4/0)	20 (16/0/4)	15/ (13/2)	34 (25/9)	30 (8/22)	–	42 (33/9)
Chordoid glioma, CNS WHO grade 2	3	–	–	–	–	3 (2/1)	–	–	–	–	–	–	–
Diffuse hemispheric glioma, H3 G34-mutant, CNS WHO grade 4	5	–	–	–	5 (5/0)	–	–	–	–	–	–	–	–
Diffuse midline glioma, H3 K27-altered, CNS WHO grade 4	9	–	–	–	8 (8/0)	–	–	–	2 (0/2)	–	–	–	–
Oligodendroglioma, IDH-mutant, and 1p/19q-codeleted, CNS WHO grade 2	18	10 (10/0)	13 (13/0)	–	–	–	7 (6/0/1)	–	–	4 (0/4)	–	–	1 (0/1)
Oligodendroglioma, IDH-mutant, and 1p/19q-codeleted, CNS WHO grade 3	29	13 (13/0)	22 (22/0)	–	1 (0/1)	–	14 (14/0/0)	–	–	6 (0/6)	–	–	5 (2/3)
Pilocytic astrocytoma, CNS WHO grade 1	25	–	–	5 (1/4)	–	–	1 (0/1/0)	–	1 (0/1)	–	–	20 (14/6)	4 (0/4)
Others	7	1 (1/0)	1 (1/0)	4 (4/0)	–	–	–	–	1 (0/1)	–	–	–	–
Blood samples	14	–	–	–	–	–	10 (0/9/1)	13 (0/12/1))	10 (0/10)	–	–	–	–
Total number of investigated samples	262	73 (61/12)	58 (49/9)	22 (7/15)	16 (13/3)	8 (2/6)	37 (20/15/2)	35 (18/12/5)	30 (13/17)	44 (25/19)	30 (8/22)	20 (14/6)	66 (41/25)

The 248 tumor samples are stratified according to glioma type. The figures in the individual columns illustrate the numbers of samples from each tumor type analysed for the respective molecular biomarker. Note that not all cases were investigated for all biomarkers by ddPCR but that cases were selected for the establishment and validation of the individual assays. Numbers in brackets: first number, cases with alteration; second number, cases without alteration; *third number, cases with non-informative SNPs. –, not analysed

### DNA preparation and quantification

DNA preparation from FFPE samples was performed by using the QIAamp DNA FFPE Tissue Kit (Qiagen, Hilden, Germany), the GeneRead DNA FFPE Kit (Qiagen), or the Maxwell® RSC FFPE Plus DNA Kit together with the Maxwell® RSC instrument (Promega, Mannheim, Germany). DNA extraction from frozen tissue samples was performed with the PureLink™ Genomic DNA Mini Kit (Life Technologies, Carlsbad, CA, USA) or by ultracentrifugation as described elsewhere [27]. Peripheral blood leukocyte DNA was extracted either with the PureLink™ Genomic DNA Mini Kit (Life Technologies) or with the Maxwell® RSC Blood DNA Kit. Extracted DNA was quantified by using the Quantus™ Fluorometer (Promega) or the Qubit system (ThermoFisher Scientific, Waltham, MA, USA).

### Droplet Digital PCR (ddPCR) analyses

Each 20 µl of PCR sample contained 1 × ddPCR supermix for probes (no dUTP) (Bio-Rad Laboratories, Munich, Germany), 900 nM forward primer, 900 nM reverse primer, and 250 nM hydrolysis probe as well as 5 µl of template DNA (≤ 5 ng/µl). The primer/probe mixes were either purchased as 20 × Droplet Digital PCR assays (Bio-Rad Laboratories) or were composed of self-designed primer and probes. Primers and dual-labelled probes were purchased from Sigma-Aldrich (Taufkirchen, Germany). Minor groove binder (MGB)-probes were ordered from Eurofins Genomics (Ebersberg, Germany). Primer and probe sequences as well as the software used for primer/probe design are listed in Additional file 1: Tables S1–S4. The ddPCR analysis was performed as described by Hindson et al. [25]. Shortly, the PCR mixture was completely transferred into the droplet generator cartridge (Bio-Rad Laboratories) and 70 µl of droplet generation oil for probes (Bio-Rad Laboratories) was added to the respective wells of the cartridge. The cartridge was covered with a gasket and placed in the QX200™ droplet generator (Bio-Rad Laboratories). The generated droplets were transferred to a 96-well PCR plate. The plate was heat-sealed with a foil seal and placed in the C1000 Touch™ thermal cycler (Bio-Rad Laboratories). PCR was performed as endpoint reaction with 40–50 cycles depending on the assay (Additional file 1: Table S5). After completion of the PCR, the 96-well plate was inserted in the QX200™ droplet reader device (Bio-Rad Laboratories) and the fluorescence of each droplet was automatically counted. The generated ddPCR data were analysed with the QuantaSoft analysis software version 1.7.4.0917 (Bio-Rad Laboratories).

### Detection of single nucleotide variants (SNVs) by ddPCR

For the detection of the *TERT* promoter mutations hg19 chr5: nucleotide 1,295,228 C > T (“C228T”) and nucleotide 1,295,250 C > T (“C250T”) we used commercially available assays from Bio-Rad Laboratories containing forward and reverse primers together with hydrolysis probes specific for the variant and for the wildtype sequence [28]. For the detection of the IDH1 p.R132 wildtype sequence and IDH1 p.R132H (c.395G > A), primer and hydrolyses probes were taken from Hirano et al. [29]. The probes for detection of the IDH1 p.R132C (c.394C > T), IDH1 p.R132G (c.394C > G), IDH1 p.R132S (c.394C > A), and IDH1 p.R132L (c.395G > T) variants were modified according to the probe used for IDH1 p.R132H. The forward and reverse primers as well as the hydrolysis probes for the detection of IDH2 p.R172 wildtype and IDH2 p.R172 variants (c.515G > A p.R172K, c.515G > T p.R172M, c.514A > T p.R172W, c.516G > T p.R172S, and c.514A > G p.R172G) were newly designed. The primer and probe sets for the detection of BRAF p.V600 wildtype and BRAF p.V600E (c.1799 T > A) sequence were taken from Hindson et al*.* [25]. The BRAF V600K (c.1798_1799delinsAA) variant specific probe was modified according to the probe used for BRAF p.V600E. Commercially available assays (Bio-Rad Laboratories) were used for the detection of the H3-3A p.K28M (c.83A > T)-mutant or the H3-3A p.K28-wildtype status. The forward and reverse primers as well as the hydrolysis probes for the detection of H3-3A p.G35-wildtype and H3-3A p.G35-mutant status (p.G35V c.104G > T, p.G35W c.103G > T, p.G35R c.103G > A, and p.G35R c.103G > C), as well as for the detection of the PRKCA p.D463-wildtype and PRKCA p.D463H (c.1387G > C)-mutant status were newly designed. All primer and probe sequences, as well as the order numbers of commercially available assays are listed in Additional file 1: Tables S1 and S2.

### Detection of copy number variations (CNVs) by ddPCR

All copy number (CN) assays were duplexed with a reference gene assay. The reference genes were located in regions that are rarely affected by copy number changes in glioma. Either commercially available ddPCR assays (Bio-Rad Laboratories) for amplification of *EGFR* exon 7 and exon 28 or a self-designed ddPCR assay for *EGFR* exon 28 were used for the detection of *EGFR* copy number changes. These enabled the concomitant investigation of amplification of the *EGFR* locus as well as the detection of a copy number difference between exons 2–8 and the remaining exons of the amplified *EGFR* gene, as corresponding to the *EGFRvIII* deletion variant [30, 31]. *NCKAP5* on 2q21.2 served as reference locus. Copy number absolute differences > 10.8 (CN *EGFR* exon 28—CN *EGFR* exon 7) according to Fontanilles et al. were considered as indicating presence of the *EGFRvIII* deletion variant [32].

For the detection of *CDKN2A* deletions on 9p21*,* a commercially available ddPCR assay (Bio-Rad Laboratories) in combination with the reference loci *NCKAP5* and *KCNS3* (2p24.2) was used. Copy number gains on chromosome 7 were determined by using eight different ddPCR assays detecting four loci on 7p (*SDK1*, *STK31*, *GLI3*, and *GBAS*) and four loci on 7q (*PDK4*, *KCND2*, *TMEM213*, and *SHH*) in combination with the reference gene *EIF4E3* on 3p13. Duplications of the *BRAF* locus at 7q34, which serves as a surrogate marker for a *KIAA1549-BRAF* fusion, were investigated using a published assay [8]. All primer and probe sequences as well as order numbers of commercially available assays are listed in Additional file 1: Tables S1 and S2.

### Detection of loss of heterozygosity (LOH) by ddPCR

To detect loss of heterozygosity on chromosomal arms 1p and 19q we established an initial set of ddPCR assays to investigate five loci containing single nucleotide polymorphisms (SNPs) (rs4648379, rs3157, rs9787003, rs1493695, rs9428240) on 1p and five loci with SNPs (rs4805965, rs6508980, rs10404903, rs10424927, rs260464) on 19q (Additional file 1: Table S3). As this set of assays turned out to be non-informative for all loci on 19q in 3 of 27 tumors (see Results section below), we established an additional set of ddPCR assays for four other SNP loci on 1p (rs2281168, rs2165194, rs4551638, rs10776720) and on 19q (rs4422091, rs7250409, rs830146, rs12463376) (Additional file 1: Table S3). Copy number losses on chromosome 10 were assessed by ddPCR assays detecting four SNP loci on 10p (rs1668538, rs10904642, rs7071770, rs1660620) and four SNP loci on 10q (rs1343043, rs4934493, rs626989, rs11816275) (Additional file 1: Table S4).

### Determination of analytical specificity and sensitivity, as well as detection limits of selected ddPCR assays

To determine the minimum amount of DNA required for reliable ddPCR-based detection of selected hotspot mutations we used tumor DNA samples carrying a *TERT* promoter “C228T” mutation (mutant allele frequency (MAF): 37.3%), a *TERT* promoter “C250T” mutation (MAF: 38.1%), an IDH1 R132H mutation (MAF: 42.6%), a BRAF V600E mutation (MAF: 22.7%), an H3-3A K28M mutation (MAF: 62.5%) or an H3-3A G35R mutation (MAF: 36.8%). Each of these mutant DNA samples was serially diluted in water, followed by the respective mutation-specific ddPCR analyses.

To determine the detection limit for these selected hotspot mutations in a wildtype DNA background, we mixed wildtype DNA samples with tumor DNA samples carrying either a *TERT* promoter “C228T” mutation (MAF 34.7%), a *TERT* promoter “C250T” mutation (MAF: 31.7%), an IDH1 R132H mutation (MAF: 42.6%), a BRAF V600E mutation (MAF: 22.7%), an H3-3A K28M mutation (MAF: 62.5%) or an H3-3A G35R mutation (MAF: 36.8%). The respective wildtype and mutant DNA samples exhibited nearly the same copies/µl value determined by ddPCR. Mutant and wildtype DNA samples were mixed in different relations resulting in samples with predefined percentages of 50%, 25%, 12.5%, 6.3%, 3.1%, 1.6%, 0.8%, and 0.4% of mutant DNA in a wildtype DNA background.

To determine the percentage of wildtype DNA contamination in a tumor sample that still allows for the detection of a *CDKN2A* homozygous deletion (defined as a gene copy number of < 0.5), we mixed DNA from the U-87 MG glioma cell line (obtained from American Type Culture Collection, ATCC, Manassas, VA), which carries a homozygous *CDKN2A* deletion [33], with *CDKN2A*-wildtype (non-deleted) tumor DNA in ratios of 90:10, 80:20, 70:30, 60:40 and 50:50, and determined the *CDKN2A* copy number by ddPCR using 25 ng of mixed DNA as template. The *CDKN2A* copy number in each mixture was calculated as followed: *CDKN2A* copy number expected = ratio of wildtype DNA x mean copy number detected for 100% wildtype DNA. The limit of blank (LoB) was determined as described by Armbruster and Pry [34].

### Detection of SNVs and CNVs by independent methods

A number of established methods were used to validate the findings obtained by ddPCR in the investigated gliomas, with the method depending on the individual biomarker. IDH mutation detection was performed by immunohistochemistry for IDH1 R132H using a mutation-specific antibody (clone H09, Dianova, Hamburg, Germany), as well as Sanger sequencing or pyrosequencing as reported [35, 36]. Hotspot mutations in *H3-3A*, *BRAF*, *PRKCA* and the *TERT* promoter were determined by either Sanger sequencing or pyrosequencing [20, 35]. In a subset of tumors, mutation data were obtained by gene panel NGS as reported before [20]. Combined deletions of 1p and 19q were assessed by microsatellite analyses for loss of heterozygosity (LOH) as reported [20, 37, 38]. DNA copy number data obtained by 850 k (EPIC) DNA methylation array technology [21] or by array-based comparative genomic hybridization (array-CGH) [39, 40] were used for validation of the ddPCR-based chromosome 7 and chromosome 10 copy number assays. *CDKN2A* gene copy number was determined by quantitative PCR using a TaqMan™ copy number assay with the reference gene *RNaseP*[20]. *EGFR* gene amplification was determined by a semiquantitative real-time PCR assay, and the presence of *EGFRvIII* was detected by reverse transcriptase PCR and/or immunohistochemistry for *EGFRvIII* as reported [30, 31]. *KIAA1549*-*BRAF* gene fusions were assessed by reverse transcription PCR analyses allowing for the detection of the three most common fusion types [41]. The primer sequences used for validation can be found in the respective references. For the investigated ddPCR assays, we determined sensitivity, specificity, accuracy, and precision in relation to the respective reference methods used for validation (Additional file 1: Table S6).

### Statistical analyses

Pearson´s correlation of calculated to detected copy numbers or allele frequencies was done using the GraphPad PRISM v8 software.

## Results

### Detection of SNVs in gliomas by ddPCR and validation by independent methods

#### TERT promoter hotspot mutations

**Fig. 1:**
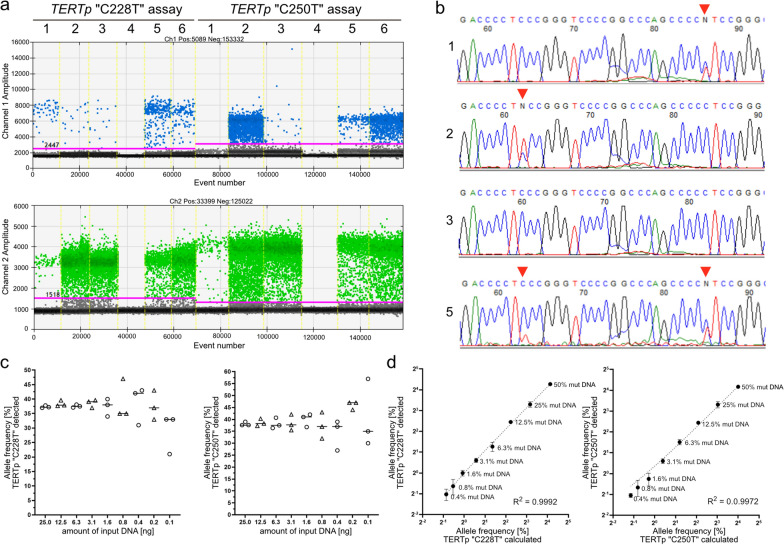
Detection of *TERT* promoter mutations in FFPE DNA extracted from gliomas using ddPCR. **a** Fluorescent intensity of the droplets after amplification of a 113 bp-fragment of the *TERT* promoter (*TERTp*) region using the ddPCR expert design assays (Bio-Rad Laboratories). The individual lanes correspond to: *1*, *TERTp* “C228T” mutation control DNA; *2*, *TERTp* “C250T” mutation control DNA; *3*, *TERTp* wildtype control DNA; *4*, no template control; *5*, glioblastoma, IDH-wildtyp, CNS WHO grade 4, and *6*, oligodendroglioma, IDH-mutant, and 1p/19q-codeleted, both with *TERTp* “C228T” and “C250T” mutations. *X axis*, number of droplets with fluorescence; *Y axis*, fluorescence intensity detected in the FAM-channel (blue dots) and HEX-channel (green dots); *pink line*, threshold; *grey dots*, droplets with background fluorescence of non-incorporated probes. **b** Validation of the two *TERT* promoter mutations in the IDH-wildtype glioblastoma using Sanger sequencing. Red arrow heads pointing to the “C250T “ (left) and “C228T” (right) mutation. The numbering of the samples corresponds to a. **c** Mutant allele frequency (MAF) was measured by ddPCR using different amounts of input FFPE DNA generated by serial dilution of samples with either a *TERTp* “C228T” (mean MAF 37.3%) or a “C250T” (mean MAF 38.1%) mutation with distilled water. **d** 25 ng of total input FFPE DNA was used for ddPCR. A *TERTp* “C228T” (mean MAF 34.7%) and a *TERTp* “C250T” DNA sample (mean MAF 31.7%) were mixed with wildtype DNA resulting in predefined templates with 50%, 25%, 12.5%, 6.3%, 3.1%, 1.6%, 0.8%, and 0.4% *TERT* promoter-mutant DNA in a *TERT* promoter-wildtype background

We analysed DNA extracted from FFPE glioma tissue samples of 73 patients for *TERT* promoter hotspot mutations at hg19 chr5 nucleotides 1,295,228 (“C228T”) and 1,295,250 (“C250T”) using commercially available ddPCR assays ([28]; Table 1). All samples were additionally analysed for these mutations by either gene panel NGS or Sanger sequencing [20]. Droplet digital PCR identified 37 “C228T” and 22 "C250T" mutations in the 73 gliomas. Two tumor samples exhibited both mutations (Fig. 1a). In one of these tumours, an IDH-mutant and 1p/19q-codeleted oligodendroglioma of CNS WHO grade 3, only the “C250T” mutation was originally detected by NGS [20]. However, a closer look at the NGS data also revealed the “C228T” mutation, which was not called before by the standard evaluation pipeline, likely due to its low mutant allele frequency (MAF) of 4.5% as determined by ddPCR. Both promoter mutations detected by ddPCR in another tumor, an IDH-wildtype glioblastoma, were independently validated by Sanger sequencing (Fig. 1b). Overall, the results of ddPCR were consistent with those obtained by NGS and/or Sanger sequencing in each of the 73 investigated gliomas.

Next, we determined the analytical sensitivity of the ddPCR assays to detect the “C228T” and “C250T” mutations in DNA extracted from FFPE glioma samples. The limit of blank (LoB) for the “C228T” variant in 20 no template samples was 0.06 copies/µl and for the “C250T” variant 0.08 copies/µl. The ddPCR assays were established by using 25 ng of FFPE DNA (as determined by fluorescence-based dsDNA quantitation) as template per ddPCR reaction. As the amount of DNA available from small tissue samples, e.g. as obtained by stereotactic biopsy, may be lower, we investigated FFPE DNA samples with a mean MAF of 37.3% (“C228T”) and 38.1% (“C250T”) at different template DNA concentrations (Fig. 1c). The mutations were still detectable with 0.1 ng of FFPE DNA as template, however, with a considerable standard deviation among the replicates. Therefore, we assumed that at least 3 ng of FFPE DNA are required for robust *TERT* promoter mutation detection by ddPCR.

Next, we mixed the “C228T” (MAF of 34.7%) or “C250T” (MAF of 31.7%) mutant DNA samples with wildtype DNA resulting in predefined DNA samples of 50%, 25%, 12.5%, 6.3%, 3.13%, 1.6%, 0.8%, and 0.4% mutant DNA (Fig. 1d). Over a large dynamic range, the expected MAF values for both *TERTp* mutations agree very well with the detected MAF values (Pearson´s R^2^ = 0.9992 for “C228T”, and R^2^ = 0.9972 for “C250T”).

The mean background allele frequency of “C228T” detected in FFPE DNA samples with a known “C250T” variant was 0.19% ± 0.22% with a highest value of 0.76% and a limit of blank (LoB) of 0.55%, and the mean background allele frequency of “C228T” detected in *TERT* promoter-wildtype samples was 0.19% ± 0.14% with a highest value of 0.52% and a LoB of 0.42%. Therefore, we decided to set the lower limit of reliable quantification for the “C228T” mutation to ≥ 1.0% MAF to minimize the detection of false-positive cases. The mean background allele frequency of “C250T” detected in FFPE DNA samples with a known “C228T” mutation was 0.23% ± 0.24%, with a highest value of 0.91% and a LoB of 0.63%. The mean background allele frequency of “C250T” detected in *TERT* promoter-wildtype samples was 0.23% ± 0.21% with a highest value of 0.83% and a LoB of 0.57%. Therefore, we concluded to set the lower limit of reliable quantification of a “C250T” mutation to ≥ 1.5% MAF to avoid detection of false positive cases. This high analytical sensitivity of *TERT* promoter mutation detection by ddPCR is of clinical relevance as it allows for mutation detection in samples with low tumor cell content, e.g. specimens from the infiltration zone of diffuse gliomas or tumor samples contaminated by marked inflammatory and reactive changes.

#### IDH1 R132 and IDH2 R172 hotspot mutations

We analysed DNA from FFPE tumor samples of 58 glioma patients for mutations involving codon 132 of *IDH1* or codon 172 of *IDH2* (Table 1). All tumors were investigated by dual probe ddPCR assays detecting one of the different IDH mutations (IDH1 R132H, R132C, R132G, R132S, R132L or IDH2 R172K, R172M, R172W, R172S, R172G) together with the respective wildtype sequence. In selected tumors (n = 21), we additionally evaluated multiplex probe assays that contained specific FAM-labelled probes for all five *IDH1* mutations or all five *IDH2* mutations together with one HEX-labelled probe for the respective wildtype sequences (Additional file 1: Table S7, Fig. S1). The multiplex assays allow for the detection or exclusion of an *IDH1* or *IDH2* mutation in just two separate reactions but do not specify the type of mutation. Overall, *IDH1* mutations were detected by ddPCR in 42 tumors while seven tumors carried *IDH2* mutations and nine tumors were IDH-wildtype. The IDH mutation status in the 58 samples was independently determined by immunohistochemistry, Sanger sequencing, pyrosequencing, and/or gene panel NGS [20, 36], which revealed concordant results in all 58 cases. The multiplex ddPCR assays identified IDH1 R132 and IDH2 R172 mutations with similar MAF values as obtained in the dual probe assays for the individual mutations (Additional file 1: Table S7).

The LoBs were exemplarily determined for the IDH1 R132H and IDH2 R172K ddPCR assays in FFPE DNA of IDH-wildtype tumors, which revealed LoBs of 0.63% MAF (IDH1 R132H assay) and of 0.29% MAF (IDH2 R172K assay), respectively. The highest background in these IDH-wildtype samples was 0.95% for IDH1 R132H and 0.36% for IDH2 R172H. Therefore, we decided to set the lower limit of reliable quantification of mutations to ≥ 1.5% MAF to avoid detection of false positive cases. A dilution series of IDH1 R132H-mutant FFPE DNA (mean MAF: 42.6%) showed that the mutation remains detectable with only 0.2 ng of template DNA, although with higher variation as compared to higher amounts of template DNA (Additional file 1: Fig. S2a). Mixing of IDH1 R132H-mutant with 42.6% MAF and *IDH1*-wildtype DNA in different relations revealed a match of expected and measured values over a wide dynamic range (Pearson´s R^2^ = 0.9998) (Additional file 1: Fig. S2a).

#### BRAF V600, H3-3A K28, H3-3A G35, and PRKCA D463 hotspot mutations

We investigated FFPE DNA from glioma tissue samples of 22 patients for BRAF V600 mutations by ddPCR (Table 1 and Additional file 1: Table S8). Six tumors exhibited a BRAF V600E and one tumor carried a BRAF V600K mutation (Additional file 1: Table S8, Fig. S3). The *BRAF* mutation status was confirmed by DNA pyrosequencing or Sanger sequencing in all 22 tumors. Investigation of a dilution series of BRAF V600E-mutant DNA (mean MAF 22.7%) showed that the mutation remains detectable by ddPCR down to 0.4 ng of template FFPE DNA, however, with increased variability between replicates (Additional file 1: Fig. S2b). Thus, if possible, more than 0.4 ng of FFPE DNA should be used for reliable ddPCR-based detection of BRAF V600E. Mixing BRAF V600E-mutant FFPE DNA (mean MAF: 22.7%) with BRAF V600-wildtype FFPE DNA in various relations revealed consistent expected and measured values over a wide dynamic range (Pearson´s R^2^ = 0.9964) (Additional file 1: Fig. S2b). A minimum mutant allele frequency of 0.13% ± 0.02 (corresponding to 0.8% mutant FFPE DNA with 22.7% MAF in a wildtype background) was detected when 25 ng of FFPE DNA were used as input (Additional file 1: Fig. S2b). The LoB of the BRAF V600E ddPCR assay determined in cases without *BRAF* hotspot mutation was 0.01%.

*H3-3A* mutations affecting the codons p.K28 or p.G35 were investigated in 16 tumors (Table 1 and Additional file 1: Table S9). Using ddPCR analyses, we identified the H3-3A p.K28M (H3 K27M) mutation in eight cases of diffuse midline glioma, H3 K27-altered, and the H3-3A p.G35R (H3 G34R) mutation in four hemispheric diffuse gliomas, H3 G34-mutant. In one tumor we detected the H3-3A p.G35V (H3 G34V) variant (Additional file 1: Fig. S4). The *H3-3A* mutational status in all tumors analysed by ddPCR was confirmed by either NGS [20], Sanger sequencing or pyrosequencing. A dilution series of H3-3A p.K28M (mean MAF 62.5%, Additional file 1: Fig. S2c) and H3-3A p.G35R (c.103G > A) (mean MAF 36.8%, Additional file 1: Fig. S2d) mutant DNA showed that the mutations remained reliably detectable by ddPCR down to 0.1 ng of template FFPE DNA for the H3-3A p.K28M variant, and down to 0.4 ng of template FFPE DNA for the H3-3A p.G35R (c.103G > A) variant. A mixture of H3-3A p.K28M (mean MAF 62.5%, Additional file 1: Fig. S2c) or H3-3A p.G35R (c.103G > A) (mean MAF 36.8%, Additional file 1: Fig. S2d) mutant FFPE DNA with H3-3A-wildtype FFPE DNA in various ratios revealed consistent expected and measured values over a wide dynamic range (Pearson´s R^2^ = 0.9996 and R^2^ = 0.9993, respectively).

For detection of the PRKCA D463H mutation, which is characteristic for chordoid gliomas [10, 11], we designed a dual probe ddPCR assay (Additional file 1: Table S1) and investigated FFPE DNA extracted from three tumors histologically classified as chordoid glioma and from five glioblastoma samples (Table 1, Additional file 1: Fig. S5). Two of the three chordoid glioma samples carried the PRKCA D463H mutation which was independently validated by Sanger sequencing (Additional file 1: Fig. S5a–c). The other six investigated glioma samples showed the PRKCA D463-wildtype sequence (Additional file 1: Fig. S5a-c).

### Detection of CNVs in gliomas by ddPCR and validation by independent methods

#### Combined deletions of chromosome arms 1p and 19q

**Fig. 2:**
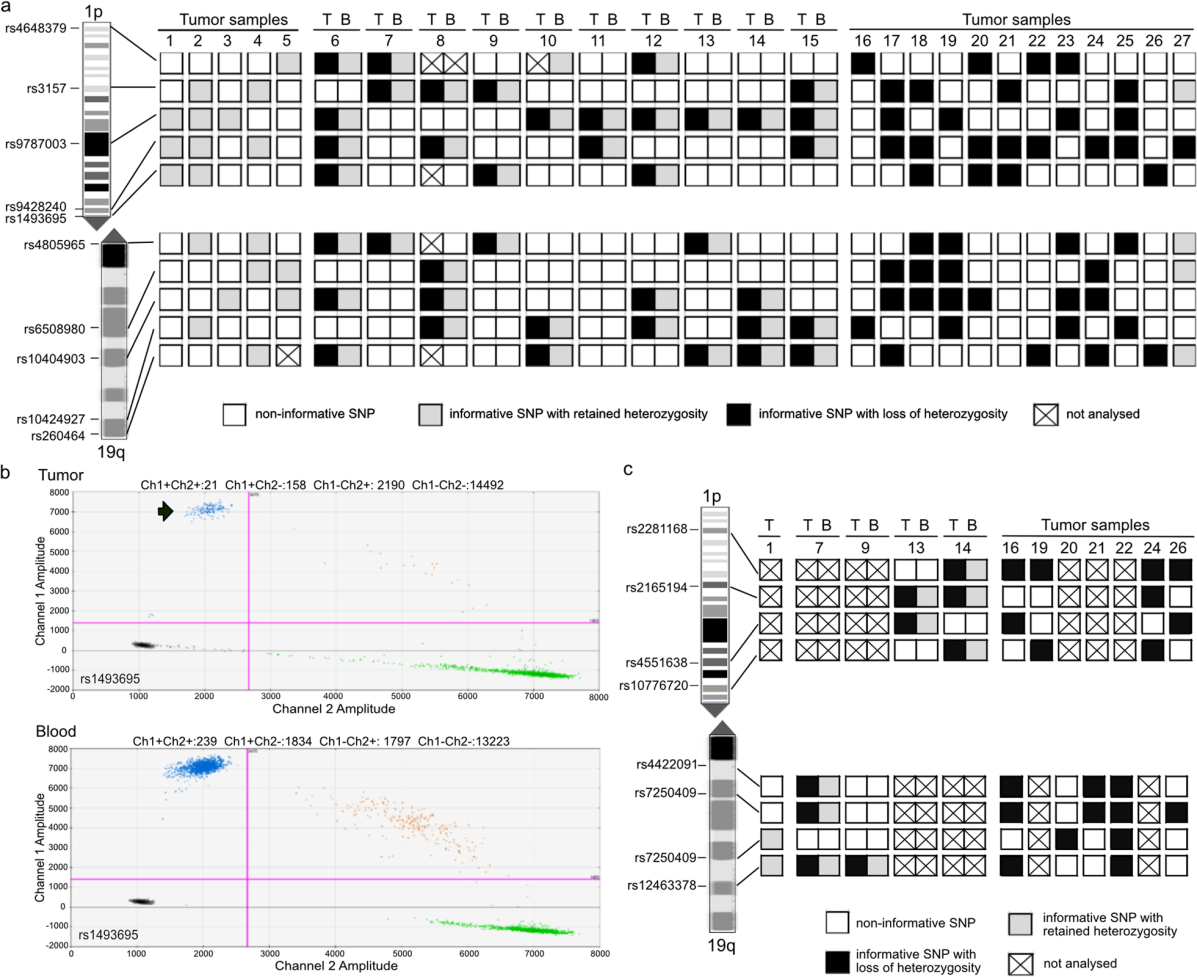
Loss of heterozygosity (LOH) on chromosomal arms 1p and 19q detected by ddPCR-based SNP analysis. **a** Patterns of ddPCR-based SNP analyses for LOH on 1p and 19q in 27 gliomas, previously shown to either have retained (tumor samples 1–5 and 27) or lost 1p and 19q (tumor samples 6–26). In ten patients with IDH-mutant and 1p/19q-codeleted oligodendrogliomas, matched DNA samples extracted from tumor tissue (T) and blood leucocytes (B) were investigated. In the remaining 17 patients, only T DNA samples were investigated. The cases 1–5 without 1p/19q codeletion were used to establish the cut-off value for the detection of LOH. Note that all patients showed one or more informative (heterozygous) SNPs on 1p, while 3 of the 27 patients lacked an informative SNP on 19q. Presence or absence of 1p/19q-codeletion in each individual case was validated by an independent method. *White rectangles*, non-informative SNP; *light grey rectangles*, informative SNP with retained heterozygosity; *black rectangles*, informative SNP with loss of heterozygosity; *crossed rectangles*, data not evaluable. **b** Exemplary presentation of a two-dimensional plot generated by the Quantasoft™ Software (Bio-Rad Laboratories). Shown are the results of the ddPCR-based analysis of SNP rs1493695 in case 9, who exhibited a LOH at this locus. *X axis*, fluorescence intensity detected in the HEX-channel (channel 2); *Y axis*, fluorescence intensity detected in the FAM-channel (channel 1); *pink line*, threshold; *grey dots*, droplets with background fluorescence; *green dots*, droplets with fluorescence detected in the HEX-channel; *blue dots*, droplets with fluorescence detected in the FAM-channel; *orange dots*, droplets with signals in both channels. The tumor-DNA showed a reduced number of droplets in the FAM-channel (arrow) compared to the number of droplets in the HEX-channel (green dots). The blood-derived DNA of this patient is heterozygous for this SNP with nearly the same number of droplets counted in the FAM- and HEX-channel. **c** Results of ddPCR analyses of additional SNPs located on 1p or on 19q in 12 selected cases, including 10 tumors with only one informative locus on one or both chromosome arms and two of the cases that were not informative at the five initially studied SNPs on 19q (see Fig. **a** above). Note that all cases demonstrated additional informative SNPs that confirmed the initial results and allowed for complete 1ß/19q copy number evaluation in the two cases that initially were not informative on 19q

We first tried to determine the 1p/19q codeletion status in gliomas by using ddPCR-based copy number analysis of eight genes mapped to chromosomal arms 1 p or 19q as reported before [42]. We applied this approach to 15 FFPE DNA samples with known retention of both chromosomal arms (Additional file 1: Fig. S6). We observed marked variations in copy number values across each of the four genes from each chromosome arm. These data suggested that 1p/19q copy number assessment relative to reference loci may not be robust enough for routine application on FFPE tumor samples. Therefore, we decided to assess 1p/19q codeletion by using ddPCR-based analysis of single nucleotide polymorphisms (SNPs) in selected loci distributed along each chromosomal arm. The advantage of this approach is that the probes specific for each of the two alleles hybridize to the same locus, i.e. amplification of a reference locus is not required for the determination of relative copy numbers. We initially selected five SNPs located on 1p and five SNPs located on 19q from the UCSC Genome Browser (Feb. 2009 (GRCh37/hg19)) that each shows a relatively high frequency of heterozygosity (Additional file 1: Table S3). For the implementation of this approach, we used FFPE-DNA of five cases without 1p/19q-codeletion (Fig. 2a, samples 1–5). The 1p/19q status of these five tumors was previously determined by gene-panel-sequencing [20]. The lowest mean value of a heterozygous SNP (rs6508980) was 47.9% ± 2.24. Thus, we set the upper cut-off value for detection of LOH at < 41.2%, i.e., mean allele frequency in 1p/19q non-codeleted tumors – 3 × standard deviation (SD). In case of uninformative, i.e., constitutionally homozygous SNPs, the mean of the highest background allele frequency (SNP rs6508980) was 0.3% ± 0.2%. Based on these allele frequencies we set the lower cut off-value for distinction of LOH from constitutional homozygosity to > 0.9% (mean allele frequency + 3 × SD).

Next, we applied these cut-off values to tumor and corresponding leukocyte DNA of 10 patients with IDH-mutant and 1p/19q-codeleted oligodendrogliomas (Fig. 2a, b, samples 6–15). The 1p/19q status of the tumors was previously established by loss of heterozygosity (LOH) analyses at six microsatellite loci on 1p and five microsatellite loci on 19q [38]. All but one of the patients were constitutionally heterozygous for at least one SNP on 1p and one SNP on 19q. The ddPCR SNP assays detected the 1p/19q codeletion in all of the nine tumors (Fig. 2a). The mean allele frequency of heterozygous loci was 49.5% ± 0.45 in the leukocyte DNA samples of these 10 patients. In 1p/19q-codeleted tumors, the allele frequency of the lost allele was reduced to values between 4.8% and 39.8%, depending on the purity of the tumor tissue samples.

Using the threshold of 0.9% and 41.2% for the less common allele, we analysed 11 independent FFPE DNA samples from gliomas with known 1p/19q-codeletion as well as one sample with known retention of 1p/19q (Fig. 2a, b, samples 16–27). Ten samples showed informative SNPs on both chromosome arms and were correctly assigned as 1p/19q-codeleted. A single sample showed LOH on 1p but remained non-informative at each of the five SNPs on 19q, and hence could not be fully evaluated for 1p/19q-codeletion. The case with previously determined retention of both chromosomal arms [20] exhibited a partial deletion of rs3157 at 1p, but retained heterozygosity at the four other informative SNP marker.

To increase the percentage of informative loci, we evaluated a set of four additional SNPs located on 1p and four additional SNPs on 19q in selected tumors, including 10 tumors with only one informative locus on one or both chromosome arms and two of the cases that were not informative at the five initially studied SNPs on 19q (case 11 was not studied due to lack of residual tumor DNA). These experiments revealed one or more additional informative loci on the investigated chromosome arms in all 12 patients, including both cases that were initially not informative on 19q, and confirmed the individual copy number alterations as detected by the reference methods (Fig. 2c).

#### Clonality of 1p/19q codeletion, TERT promoter mutation and IDH mutation in IDH-mutant and 1p/19q-codeleted oligodendrogliomas

The quantitative determination of allele frequencies for SNVs and SNPs by ddPCR allows for the analysis of clonality versus subclonality of different genetic alterations in a given tumor DNA sample. We exemplified this by comparing mutant allele frequencies of *IDH1* or *IDH2* and the *TERT* promoter, as well as deleted allele frequencies on 1p and 19q in a cohort of 20 IDH-mutant and 1p/19q-codeleted oligodendrogliomas (Additional file 1: Fig. S7; Table S10). We calculated the expected deleted allele frequency at the informative SNPs on 1p and 19q based on the MAF obtained for the *TERT* promoter mutation (Additional file 1: Fig. S7a, Table S10a) or the IDH mutation (Additional file 1: Fig. S7b, Table S10b). Three of the 20 oligodendrogliomas were *TERT* promoter wildtype. In 19/20 tumors, the IDH MAFs and in 16/17 tumors the *TERT* promoter MAFs corresponded well to the deleted allele frequencies on 1p and 19q, suggesting clonality of each of the three alterations in these tumors. In one tumor, however, the percentages of the lost alleles on 1p and 19q were more than 10% lower than the expected values calculated from the *TERT* and *IDH1* MAFs, possibly suggesting 1p/19q codeletion as the initial clonal event in this particular tumor.

#### Chromosome 10 deletion

**Fig. 3:**
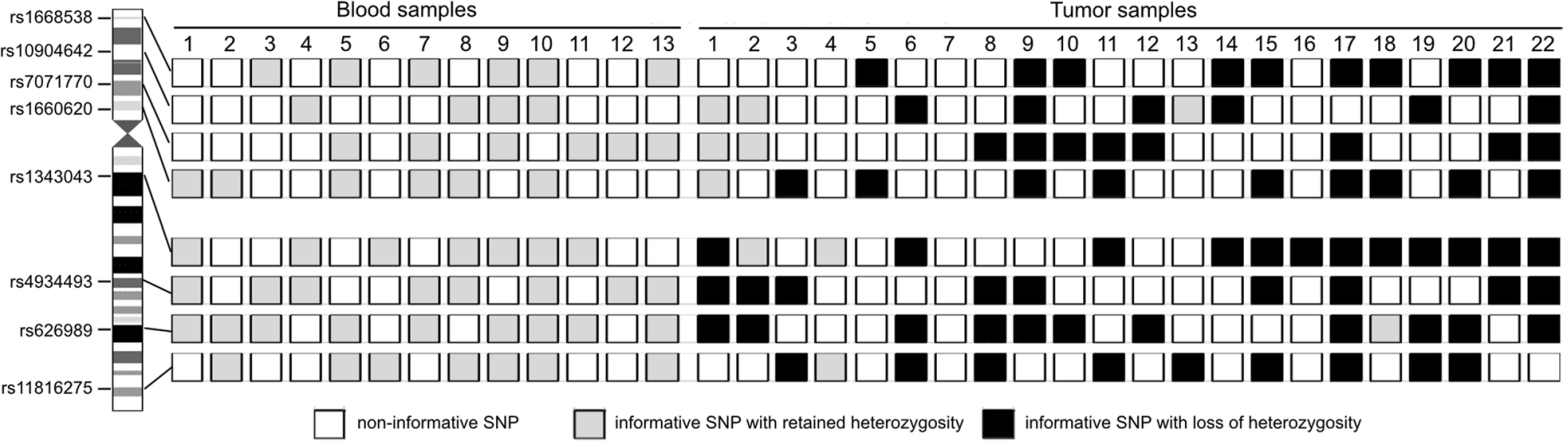
Patterns of ddPCR-based SNP analyses for loss of heterozygosity on chromosome 10 in 13 control blood samples and 22 selected tumor samples, previously demonstrated to have losses on 10q only (tumor samples 1- 3), no losses on 10 (tumor sample 4), or losses on both 10p and 10q (tumor samples 5–22). *White rectangles*, non-informative SNP; *light grey rectangles*, informative SNP with retained heterozygosity; *black rectangles*, informative SNP with loss of heterozygosity. *Tumor sample 1*, astrocytoma, IDH-mutant, CNS WHO grade 2; *Tumor sample* *2*, astrocytoma, IDH-mutant, CNS WHO grade 4; *Tumor samples 3–22*, glioblastoma, IDH-wildtype, CNS WHO grade 4. The majority of samples showed at least one informative (heterozygous) SNP on each chromosomal arm, except for 3 samples that were not informative at the SNPs on 10p, one sample that was not informative at the SNPs on 10q, and one sample that was not informative at the 8 SNPs at both chromosomal arms. Presence or absence chromosome 10 losses in the individual tumor cases were independently validated by other methods (see text)

For the detection of chromosome 10 losses in gliomas, we designed SNP-based ddPCR assays as described above for 1p/19q codeletion. We selected four SNPs in loci on 10p (rs1668538, rs10904642, rs7071770, rs1660620) and four SNPs in loci on 10q (rs1343043, rs4934493, rs626989, rs11816275) (Additional file 1: Table S4). We investigated constitutional (leukocyte) DNA from 13 patients as well as tumor DNA with known chromosome 10 deletion status [39] from 22 patients (18 from FFPE samples and four from fresh-frozen samples) (Fig. 3). Applying the cut-off values experimentally determined for the 1p/19q codeletion analysis by SNP-based ddPCR, 12 of the 13 constitutional DNA samples were informative at one or more loci on each chromosomal arm, while one sample was informative on 10q but homozygous for all four loci on 10p. In 15 of 22 cases ddPCR analyses detected the losses on chromosome 10 previously determined by array-based comparative genomic hybridization [39]. One sample was homozygous (not informative) for all investigated SNP markers. One sample without LOH on chr10 retained both informative SNP markers on 10q, but was not informative for the four SNPs on 10p. Two samples were informative for SNP markers on either 10p or 10q, but uninformative for the four SNP markers on the other chromosomal arm. In two cases one of the heterozygous SNP markers (rs10904642 and rs626989, respectively) showed MAF values slightly crossing the threshold of > 41.2%. A closer look at the allele frequency of the *TERTp* mutation detected in both tumors revealed a low frequency of the mutated allele (8.9% and 14.6%, respectively) pointing to a low tumor cell content in both tumor samples. This observation might explain that the MAF values of the SNP markers in both tumor cases were slightly below or above the threshold. In one case we could validate a loss on 10q, but additionally detected LOH at the informative SNP marker rs1660620 on 10p, indicating 10p losses that were not detected previously by array-CGH [39].

In 13 IDH-wildtype glioblastomas with chromosome 10 losses and *TERT* promoter mutations (10 tumors with “C228T”, 3 tumors with “C250T”) we compared the *TERT* promoter MAFs with the deleted allele frequencies on chromosome 10 as detected by ddPCR (Additional file 1: Fig. S7c). The allele frequencies matched well to each other, with minor differences between detected and calculated values ranging between 0.5% and 6.2% (Additional file 1: Table S11), except for one case with a difference of 15.0%, however, overall consistent with clonality of *TERT* promoter mutation and chromosome 10 losses in these tumors.

#### Chromosome 7 gain

**Fig. 4:**
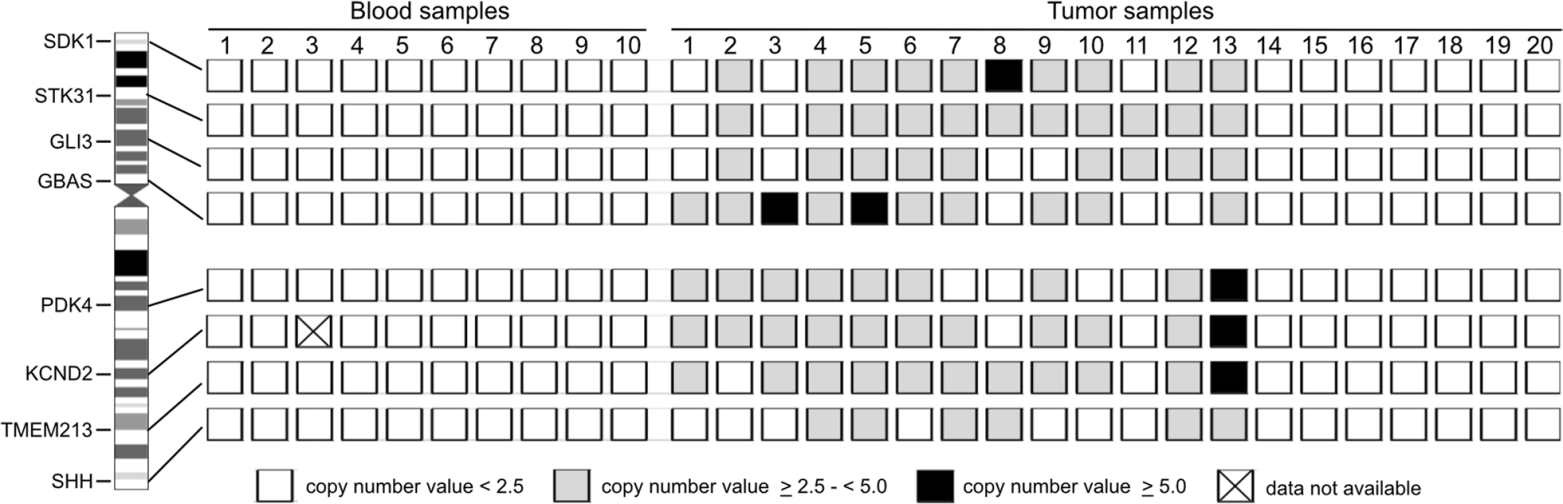
Gene copy number changes on chromosome 7 detected by ddPCR-based copy number analysis in 10 control blood samples and 20 tumor samples, previously shown to have a chromosome 7 gain (tumor samples 1–13) or a balanced chromosome 7 status (tumor samples 14–20). The threshold for a copy number gain at an individual genomic locus was set to ≥ 2.5 according to Crespo et al*.* [43]. *Tumor samples 1–15*, glioblastoma IDH-wildtype, CNS WHO grade 4; *Tumor sample 16*, astrocytoma, IDH-mutated, CNS WHO grade 4; *Tumor samples 17–18*, diffuse midline glioma, H3 K27-altered; *Tumor sample 19*, pilocytic astrocytoma, CNS WHO grade 1; *Tumor sample 20*, others (atypical teratoid/rhabdoid tumor). *White rectangles*, copy number value < 2.5; *light grey rectangles*, copy number value ≥ 2.5 and < 5.0 (low-level copy number gain); *black rectangles*, copy number value ≥ 5.0 (high-level copy number gain/gene amplification); *crossed rectangle,* data not available. None of the control blood samples displayed evidence of gains of whole chromosome 7 indicative of trisomy 7. The results obtained by ddPCR for chromosome 7 gain by ddPCR were independently validated by other methods. Note that individual tumors demonstrated evidence for focal high-level copy number changes on the background of whole chromosome 7 gain

For the detection of copy number gains on chromosome 7 we designed primer/probe assays with FAM-labelled probes specific for the detection of four genes (*SDK1, STK31, GLI3, GBAS*) mapping to 7p and four genes (*PDK4, KCND2, TMEM213, SHH*) mapping to 7q. A primer/probe assay with a HEX-labelled probe specific for *EIF4E3* on 3p13 was used as reference locus (Additional file 1: Table S2). We investigated 10 constitutional (leukocyte) DNA samples, and 20 tumor samples, including four low-grade gliomas, one IDH-mutant astrocytoma, CNS WHO grade 4, and 15 IDH-wildtype glioblastomas for copy number (CN) alterations of chromosome 7 (Fig. 4). The mean CN determined for the constitutional DNA samples was 1.98 ± 0.04. Following the publication by Crespo et al. [43], we used a threshold for chromosome 7 low-level gain of ≥ 2.5, while CN gains of ≥ 5.0 were considered as high-level gain/amplification of the respective genomic region. Nine of the 16 investigated glioblastomas showed low-level gains for at least six of the eight investigated loci on chromosome 7. One of these tumors additionally showed a focal high-level gain of *GBAS* on 7p11.2 that was found to be coamplified with *EGFR* in this case (Fig. 4). Three glioblastomas showed focal gains involving loci on 7p as well as on 7q. One glioblastoma exhibited only a gain for *STK31* on 7p15.3 and *GLI3* on 7p14.1. The remaining three glioblastomas and the four low-grade gliomas showed no chromosome 7 gains. The data obtained by ddPCR in the 20 tumor samples were validated by microarray-based copy number profiling using 850 k DNA methylation arrays [44] or by array-CGH [39, 40]. Ten of the 16 glioblastomas were also analysed by ddPCR for chromosome 10 deletions. In total, six of these 10 glioblastomas demonstrated chromosome 10 deletions by ddPCR analysis previously verified by array-CGH [39], and therefore showed a + 7/-10 signature by ddPCR that was validated with independent methods.

#### EGFR gene amplification

**Fig. 5:**
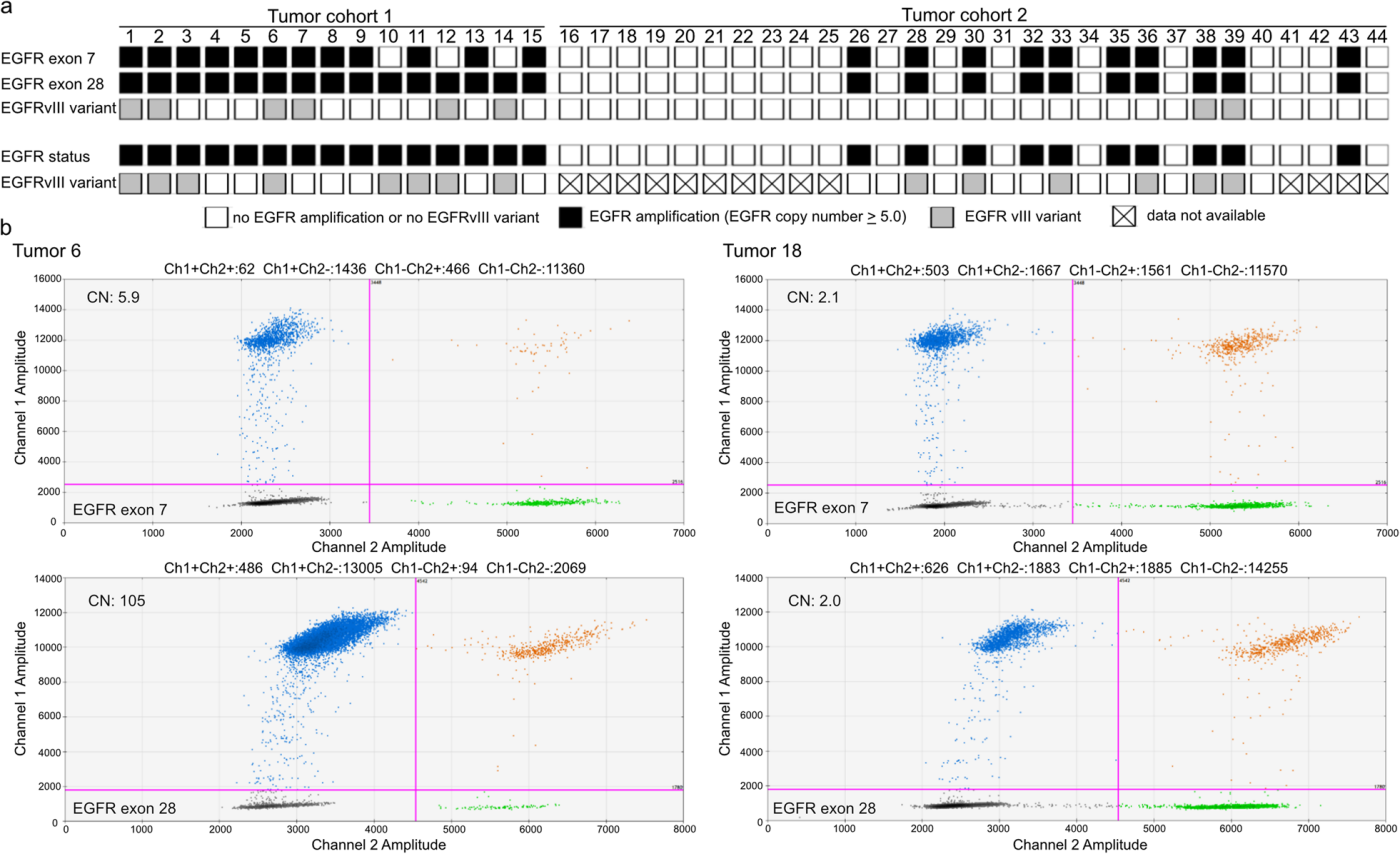
Detection of focal copy number changes of the *EGFR* gene on chromosome 7p11.2**.** Copy number analysis was done by ddPCR using a PrimePCR™ ddPCR copy number assay (Bio-Rad Laboratories) for the analysis of exon 7 and a newly designed ddPCR assay for exon 28 copy number changes. **a** Schematic representation of *EGFR* copy number changes. *Upper three rows*, Results of *EGFR* amplification analysis by ddPCR assays. *Lower two rows*, Data obtained by independent methods. Tumor cohort 1 corresponds to selected cases with known *EGFR* copy number status before ddPCR analysis for *EGFR* amplification. In tumor cohort 2, results obtained by ddPCR were validated afterwards by other methods. *Tumor samples 1–15, 26–44*, glioblastoma, IDH-wildtype, CNS WHO grade 4 (samples 1 and 2, 11 and 12 as well as 13 and 14 are pairs of primary and recurrent tumor); *Tumor samples 16–19*, oligodendroglioma, IDH-mutant, and 1p/19q codeleted, CNS WHO grade 2; *Tumor samples 20–25*, oligodendroglioma, IDH-mutant, and 1p/19q codeleted, CNS WHO grade 3. *White rectangles*, no *EGFR* amplification (*EGFR* copy number < 5.0); *black rectangles*, *EGFR* gene amplification (*EGFR* copy number ≥ 5.0); *grey rectangles*; *EGFRvIII* variant; *crossed rectangles*, data not available. **b** Exemplary presentation of two-dimensional plots generated by the Quantasoft™ Software. Shown are the results of the ddPCR-based analysis of *EGFR* exon 7 and exon 28 copy number changes in the tumor samples 6 and 18. *X axis,* fluorescence intensity detected in the HEX-channel (channel 2); *Y axis*, fluorescence intensity detected in the FAM-channel (channel 1); *pink line*, threshold; *grey dots*, droplets with background fluorescence; *green dots*, droplets with fluorescence detected in the HEX-channel; *blue dots*, droplets with fluorescence detected in the FAM-channel; *orange dots*, droplets with signals in both channels. Tumor sample 6 exhibited a high-level *EGFR* amplification and the *EGFRvIII* variant. Note the high number of blue dots in channel 1 for *EGFR* exon 28 compared to the green dots in channel 2 for the reference gene, whereas the number of blue dots in channel 1 for exon 7 are markedly lower than for exon 28. Tumor sample 18 showed no *EGFR* copy number change with nearly the same number of droplets for exon 7 and exon 28 in both channels

*EGFR* gene copy number was first assessed by ddPCR in 15 IDH-wildtype glioblastomas with previously demonstrated *EGFR* amplification [20] (Fig. 5a, samples 1–15). Eight of these tumors additionally carried the *EGFRvIII* deletion (Fig. 5a, b). We first used two commercially available PrimePCR™ ddPCR copy number assays (Bio-Rad Laboratories Laboratories, Additional file 1: Table S2) for *EGFR* exons 7 and 28. With these assays, we validated *EGFR* amplification in all 15 tumors, however, could not reliably identify the presence of the *EGFRvIII* variant by detecting higher copy numbers for exon 28 compared to exon 7 (Fig. 5a). This might be due to the larger amplicon size for *EGFR* exon 28 of 88 base pairs (bp) compared to 64 bp for exon 7. Therefore, we designed a novel ddPCR assay for *EGFR* exon 28 with an amplicon size of 64 bp (Additional file 1: Table S1). This allowed for detection of higher copy numbers for exon 28 compared to exon 7 in 12 of 13 tumors investigated with both *EGFR* exon 28 assays and resulted in the identification of one more case with *EGFRvIII* that was missed before (Additional file 1: Table S12, tumor no. 8). However, the *EGFRvIII* variant was not detected in the three glioblastomas shown to carry this alteration by other methods (Fig. 5a).

In a next step, we examined the *EGFR* copy number status in 10 lower grade gliomas for which we expected no *EGFR* copy number increase (Fig. 5a, samples 16–25). The mean *EGFR* copy number values in these samples were 1.9 ± 0.3 for exon 7 and 2.0 ± 0.2 for exon 28 (Fig. 5b). Then, we analysed tumor DNA samples from 19 IDH-wildtype glioblastomas with known *EGFR* status (Fig. 5a, samples 26–44). In 10 of the 19 samples, ddPCR identified *EGFR* amplification with a copy number range of 6.1—151.0 for exon 28, while the remaining nine samples lacked *EGFR* amplification. Subsequent analyses by real-time PCR-based copy number analyses confirmed the *EGFR* status determined by ddPCR in each of the 29 tumors. Two of the *EGFR*-amplified samples showed lower copy number gains for *EGFR* exon 7 compared to exon 28 indicating the *EGFRvIII* variant. Using immunohistochemistry for *EGFRvIII* corroborated the presence of *EGFRvIII*-positive tumor cells in these two tumors as well as four additional glioblastomas with *EGFR* amplification. These results indicate that ddPCR detects *EGFR* amplification in glioblastomas with high sensitivity and specificity (Additional file 1: Table S6). However, ddPCR-based detection of *EGFRvIII* by comparing copy number levels at *EGFR* exon 7 and exon 28 in *EGFR*-amplified tumors has lower sensitivity as compared to *EGFRvIII* immunohistochemistry (Additional file 1: Table S6).

#### BRAF duplication

We used the ddPCR approach reported by Appay and co-workers [8] to investigate DNA extracted from FFPE samples of pilocytic astrocytomas of 20 patients for *BRAF* duplication as a surrogate marker for *KIAA1549-BRAF* fusions (Additional file 1: Fig. S8). All cases were previously analysed by reverse transcriptase real time-PCR for the presence of *KIAA1549-BRAF* fusion, and 13/20 tumors exhibited fusions at the mRNA level. We adopted the primer and probe sequences as well as the assay conditions and the threshold for copy number duplication (CN > 2.25) from Appay et al. [8]. Using these parameters, we observed a copy number gain > 2.25 in all 13 cases with *KIAA1549-BRAF* fusions and in one pilocytic astrocytoma lacking detectable *KIAA1549-BRAF* fusions by RT-PCR analysis (Additional file 1: Fig. S8). We re-analysed this tumor by using an ARCHER® FusionPlex NGS panel and were able to validate a fusion of *KIAA1549* exon 14 to *BRAF* exon 8 in this case (Additional file 1: Fig. S9). This fusion is not covered by the primer pairs commonly used for RT-PCR-based fusion analysis [41]. Taken together, our data independently validate those reported by Appay et al*.* [8] and support ddPCR-based detection of *BRAF* duplication in FFPE DNA as a sensitive surrogate marker for *KIAA1549-BRAF* fusions.

#### CDKN2A homozygous deletion

**Fig. 6:**
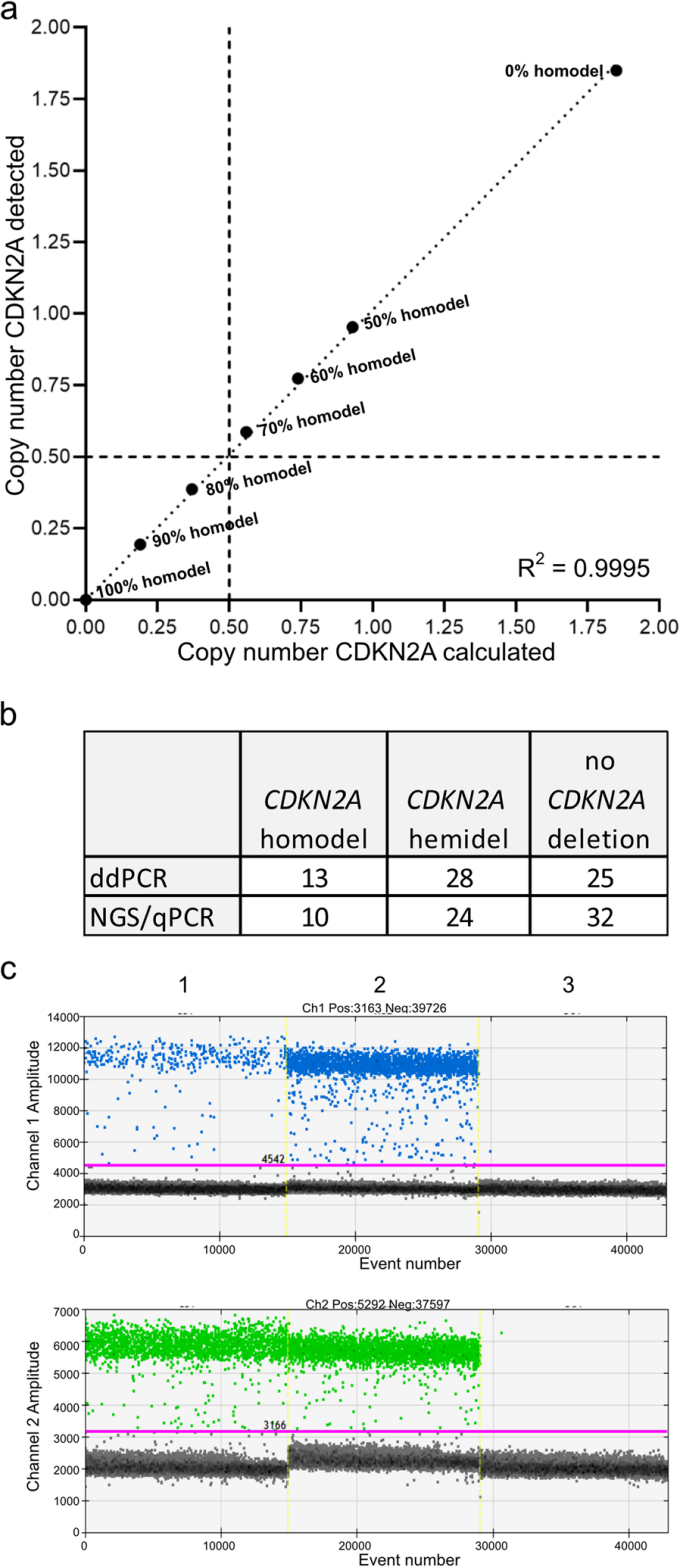
Detection of *CDKN2A* tumor suppressor gene deletions in gliomas by ddPCR-based copy number analysis. **a** Results of Pearson´s correlation analysis of the calculated *CDKN2A* copy number compared to the detected copy number (CN) in mixtures of U87-MG DNA, which has a homozygous deletion of *CDKN2A*, and a control DNA that retained both *CDKN2A* copies. The copy numbers of the undiluted DNA were determined by ddPCR (100% U87-MG DNA: mean CN 0.0; control-DNA: mean CN 1.85 ± 0.02). **b** Results of *CDKN2A* copy number analyses using a PrimePCR™ ddPCR copy number assay (Bio-Rad Laboratories) in 66 glioma samples in comparison to other methods (NGS or qPCR). *homodel,* homozygous deletion; *hemidel*, hemizygous deletion; *ddPCR*, droplet digital PCR; *NGS*, gene panel next generation sequencing [20]; *qPCR*, semiquantitative real-time PCR. Note that ddPCR-based analysis identified three tumors with homozygous *CDKN2A* deletion that were considered as showing hemizygous deletions with the other methods. **c** Fluorescent intensity of the droplets after duplex-PCR using a PrimePCR™ ddPCR copy number assay (Bio-Rad Laboratories) for amplification of a 66 bp-fragment of the *CDKN2A* locus (*upper row*) together with self-designed primers for amplification of a 86 bp-reference gene locus (*lower row*) (*NCKAP5*, see Additional file 1: Table S2). *X axis*, number of droplets with fluorescence; *Y axis*, fluorescence intensity detected in the FAM-channel (Channel 1, blue dots) and HEX-channel (Channel 2, green dots); *pink line*, threshold; *grey dots*, dots with background fluorescence of non-incorporated probes; *Lane 1*, the IDH-mutated astrocytoma, CNS WHO grade 4, exhibited a homozygous deletion of the *CDKN2A* locus (CN < 0.5). Note that the number of blue droplets are markedly lower than the number of green droplets indicating a homozygous *CDKN2A* deletion. The few remaining blue droplets were caused by *CDKN2A* non-deleted cells. *Lane 2*, the glioblastoma, IDH-wildtype, CNS WHO grade 4, retained both copies of *CDKN2A* and showed nearly the same numbers of droplets in channel 1 and channel 2. *Lane 3*, no template control

The ddPCR-based assay for *CDKN2A* copy number analysis was evaluated by mixing DNA from *CDKN2A* homozygously deleted U87-MG cells with tumor DNA from a glioma that had retained both *CDKN2A* copies by ddPCR analysis (copy number: 1.85 ± 0.02). Both DNA samples were mixed in ratios of 90:10, 80:20, 70:30, 60:40, and 50:50. 25 ng of each DNA mixture was used for ddPCR (Fig. 6a). The calculated copy numbers of the respective mixtures correlated well with the experimentally detected copy numbers across all mixtures (Pearson’s R^2^ = 0.9995). As a threshold for detection of *CDKN2A* homozygous deletion in a tumor DNA sample, we used a copy number value of 0.5, which could reliably detect a homozygous deletion in a background of less than 25% non-deleted cells, i.e., a tumor cell content of ≥ 75% (Fig. 6a). Copy number values of ≥ 0.5 and < 1.5 were regarded as suggestive for hemizygous deletions.

To validate the *CDKN2A* ddPCR assay in glioma tissue samples, we investigated 66 gliomas of different types with known *CDKN2A* copy number status determined by NGS [20] and/or a TaqMan™ copy number assay with the reference gene *RPPH1*, including 10 cases with homozygous *CDKN2A* deletion (Fig. 6b, c, Additional file 1: Table S13). All 10 cases were also detected by ddPCR as being homozygously deleted. In addition, we detected homozygous *CDKN2A* deletion by ddPCR in three more tumors that appeared as carrying a hemizygous deletion using the TaqMan™ copy number assay. All three cases exhibited a copy number close to the threshold of 0.5 either slightly lower than the threshold using ddPCR or slightly higher than the threshold using the TaqMan™ copy number assay. Among the remaining 53 tumors, 28 tumors showed reduced copy number values suggestive of hemizygous *CDKN2A* deletion by ddPCR, 21 (75%) of which also showed correspondingly reduced *CDKN2A* copy numbers in the TaqMan™ copy number assay, seven tumors had calculated copy number values above the threshold of 1.5 by using the TaqMan™ copy number assay. Strikingly, the cases with incongruent results always exhibited higher copy number values using the TaqMan™ copy number assay compared to ddPCR. In total, the results of ddPCR were corroborated by TaqMan™ copy number or NGS analyses in 56 of 66 (85%) cases.

### Workflow for ddPCR-based molecular diagnostics of gliomas in the routine setting

Following the validation of the ddPCR-based assays reported above, we decided to translate the technology into routine molecular diagnostic application. We therefore established standard operating procedures for each assay that are being followed in routine diagnostics. The general work flow for the ddPCR-based molecular diagnostics is depicted in Additional file 1: Fig. S10, which shows that each ddPCR analysis can be accomplished within one working day, starting from extraction of DNA and ending with the evaluation of the ddPCR results. Moreover, all investigated ddPCR assays detected the respective diagnostic alterations with high sensitivity, specificity, accuracy, and precision in relation to the individual reference methods (Additional file 1: Table S6).

## Discussion

We report on the application and validation of ddPCR-based analyses for the detection of diagnostic molecular biomarkers in gliomas. In total, ddPCR-based assays for the following molecular biomarkers were evaluated: *IDH1* codon 132 mutations, *IDH2* codon 172 mutations, *H3-3A* codon 28 mutation, *H3-3A* codon 35 mutations, *TERT* promoter mutations, *BRAF* codon 600 mutations, *PRKCA* codon 463 mutation, 1p/19q codeletion, chromosome 7 gain and chromosome 10 loss (+ 7/-10), *EGFR* amplification, *CDKN2A* homozygous deletion, and *BRAF* duplication as surrogate marker for *KIAA1549-BRAF* gene fusions. Sensitivity, specificity, accuracy and precision of the respective ddPCR-assays was determined in relation to established methods for each diagnostic alteration. Collectively, the reported assays cover nearly all of the diagnostic biomarkers required for the classification of adult-type diffuse gliomas according to the WHO classification 2021 [1]. At present, only *ATRX* mutation is a biomarker that cannot be assessed in a reasonable manner by ddPCR due to the widespread distribution of mutations across the entire gene [45]. However, loss of nuclear ATRX expression is easily detectable by immunohistochemistry to distinguish IDH-mutant astrocytomas from IDH-mutant and 1p/19q-codeleted oligodendrogliomas [46, 47].

Our data are supporting and extending recent reports on the use of ddPCR assays to detect mutations in glioma tissue samples, including IDH mutations [29, 48, 49], *TERT* promoter mutations [49, 50], H3-3A mutations [51], and *BRAF* duplication [8]. In addition, successful use of ddPCR for detection of *FGFR1* mutations and internal tandem duplication of the tyrosine-kinase domain in dysembryoplastic neuroepithelial tumors has been reported [51, 52]. Collectively, our findings and the published data clearly indicate ddPCR-based analyses as a robust, sensitive and specific method that enables the detection of even low-abundance mutations in a high background of wildtype sequence. Our study indicates that MAFs of as low as 1%—1.5% can be reliably detected by ddPCR in DNA extracted from FFPE tumor specimens, a finding in line with previous studies, which indicated similar or even lower limits of detection depending on the individual assay and the amount and type (DNA from FFPE or frozen tissue samples) of template DNA [25, 28, 29]. In our experiments, we typically used 20–30 ng of template FFPE DNA as input for ddPCR analysis, however, we show that lower amounts of down to 1 ng of template DNA may still allow for reliable mutation detection. Again, this finding is in line with data from other studies using low amounts of template DNA, e.g. for detection of glioma-associated mutations in cell-free DNA (cfDNA) or exosomal RNA extracted from cerebrospinal fluid (CSF) samples [53–55] or cfDNA in blood serum [50]. Overall, analytical sensitivity of ddPCR is considerably higher compared to Sanger sequencing, which is commonly used as the diagnostic standard method, as demonstrated in our dilution series for selected IDH mutations and *TERT* promoter mutations, as well as in another study for *BRAF* and *TERT* promoter mutations in melanoma FFPE tissues [56]. The increased analytical sensitivity of ddPCR is not associated with reduced specificity [48, 49, 57], as also demonstrated in our study by the validation of ddPCR results with other methods.

A specific advantage of ddPCR compared to qualitative or semiquantitative approaches is the generation of absolutely quantitative data on mutant allele frequencies and DNA copy numbers [26]. This allows for the definition of cut-off values for reliable detection of mutations, even at low MAFs, and the distinction of different levels of copy number variations, such as the diagnostically relevant distinction between low-level gain from high-level gain (amplification) of the *EGFR* gene in IDH-wildtype glioblastomas, and homozygous from hemizygous deletion of the *CDKN2A* gene in IDH-mutant astrocytomas. Moreover, the quantitative ddPCR data enable the distinction of clonal (early) from subclonal (later) genetic alterations by comparing allele frequencies in a given tumor sample. Thereby, we found that chromosome 10 deletion and *TERT* promoter mutation are both clonal in most IDH-wildtype glioblastomas, which is in line with previous whole genome sequencing data showing *TERT* promoter mutations as clonal events in the majority of these tumors, while subclonal *TERT* promoter mutations were found in a subset of IDH-wildtype glioblastomas [58]. In IDH-mutant and 1p/19q-codeleted oligodendrogliomas, we found that IDH mutation, *TERT* promoter mutation, and 1p/19q codeletion are clonal alterations in the vast majority of tumors. In addition, ddPCR-based detection of early (clonal) mutations such as IDH mutation, *TERT* promoter mutation and chromosomal losses like 1p/19q codeletion or chromosome 10 deletion may serve as an objective means to assess tumor cell content in a given tissue sample more accurately than microscopic evaluation. This might be of relevance to assure sufficient tumor cell content for correct evaluation of deletions, e.g. the distinction of homozygous from hemizygous *CDKN2A* deletion in an IDH-mutant astrocytoma.

In comparison to microsatellite-based LOH analyses for 1p/19q-codeletion or loss of chromosome 10, the SNP-based ddPCR-assays reported in this study have the advantage of not requiring additional investigation of constitutional (leukocyte) DNA as reference. A limitation, however, is the lower degree of heterozygosity at the SNP loci compared to highly polymorphic microsatellite loci, which in a minor fraction of patients may reveal non-informative data due to homozygosity of all markers on the respective chromosome arm. As demonstrated here for 1p/19q codeletion testing, this issue can be addressed by investigation of additional SNPs in such cases, which also substantiated the findings in our cases initially showing only single informative SNPs on either chromosome arm.

An important advantage of the ddPCR technology relates to the fast generation of results, with the time required from extraction of DNA to completed evaluation of the PCR results being less than 8 h, hence providing diagnostic molecular results within a single day. Thus, the approach can considerably speed up the routine diagnostic process for gliomas, in particular when compared to NGS and microarray-based methylation profiling that typically require several working days for data generation [44]. On the other hand, however, NGS approaches allow for a more comprehensive detection of mutational landscapes [18–20], and DNA methylation profiling enables epigenetic tumor classification and detection of genome-wide copy number aberrations [21], which both cannot be accomplished by ddPCR analyses. Hence, each of these molecular diagnostic approaches provides distinct advantages that complement each other in the routine diagnostic setting.

A potential limitation of ddPCR relates to the requirement of multiple PCR reactions to detect different types of mutations in a given gene, e.g. at *IDH1* codon 132 or *IDH2* codon 172, or several loci across chromosomal arms, e.g. for 1p/19q codeletion or + 7/-10 testing. For example, the amount of tumor DNA for the 20 ddPCR runs needed for IDH mutation and 1p/19q codeletion analyses sums up to 500 ng when 25 ng DNA is used for each individual run. In case of limited DNA availability, e.g. due to small tissue samples, this may require reduction of DNA input or the use of multiplex assays [51] or drop-off ddPCR approaches [59] for joint analyses of more than one mutation in a single run. Nevertheless, the requirement of DNA template for analyzing multiple biomarkers by ddPCR is higher when compared to parallel analysis of multiple genes by NGS-based gene panel sequencing [18–20].

A further limitation concerns the ddPCR assay used for *EGFR* copy number analysis. While this assay reliably detected *EGFR* amplification, i.e., one of the relevant molecular biomarkers for IDH-wildtype glioblastoma [60], its analytical sensitivity for additional detection of the *EGFRvIII* variant in *EGFR*-amplified glioblastomas was lower compared to RT-PCR analysis and immunohistochemistry. As previously reported [30, 31], this relates to the fact that *EGFRvIII* may be restricted to single tumor cells or minor tumor cell fractions, which do not result in intragenic copy number differences detectable at the DNA level by ddPCR or other DNA-based approaches such as gene panel NGS and multiplex-ligation probe amplification (MLPA).

In summary, our findings and those reported by other groups [8, 28, 29, 32, 42, 48–52] clearly support ddPCR-based analyses as a valuable approach for robust, rapid and quantitative detection of glioma-associated, diagnostically relevant SNVs and CNVs in the routine diagnostic setting. The glioma-tailored ddPCR assays reported to date may be continuously complemented by novel assays for the detection of further diagnostic alterations in gliomas and other primary brain tumor types.

## Supplementary Information

**Additional file1**. **Table S1** Overview of the primer and probe sequences used for the individual ddPCR assays in this study including both previously published and newly designed assays. **Table S2** Commercially available assays used for ddPCR-based single nucleotide variant (SNV) and copy number variation (CNV) analyses of certain biomarkers. **Table S3** Primers and probes used for ddPCR-based single nucleotide polymorphism (SNP) analysis on chromosomal arms 1p and 19q. **Table S4** Primers and probes used for ddPCR-based SNP analysis on chromosome 10. **Table S5** Thermocycler conditions used for ddPCR assays. **Table S6** Sensitivity, specificity, accuracy, and precision of each ddPCR assay investigated in relation to the respective method used for validation. **Table S7** Detection of *IDH1* and *IDH2* mutations using duplex and multiplex ddPCR assays. **Table S8** Detection of BRAF V600E and BRAF V600K mutations using duplex ddPCR assays. **Table S9** Detection of H3-3A p.K28M and H3-3A p.G35R mutations using duplex ddPCR assays. **Table S10** Comparison of the experimentally detected mean allele frequency (AF) of deleted SNP loci on 1p and 19q versus the AF calculated from the mutant allele frequency (MAF) of *TERTp* (a) or *IDH1/2* (b) mutations. **Table S11** Comparison of the experimentally detected mean allele frequency (AF) of deleted SNP loci on chromosome 10 versus the AF calculated from the mutant allele frequency (MAF) of *TERTp* mutations. **Table S12** Detection of the *EGFRvIII* variant using ddPCR as well as comparison of a commercially available PrimePCR™ ddPCR (a) and a self-designed (b) copy number assay for EGFR exon 28. **Table S13** Determination of *CDKN2A* copy number in 66 glioma samples using a PrimePCR™ ddPCR CDKN2A copy number assay. **Fig. S1** Detection of *IDH1* and *IDH2* hotspot mutations in FFPE DNA using ddPCR. **Fig. S2** Sensitivity of ddPCR to detect IDH1 R132H (a), BRAF V600E (b) as well as H3-3A p.K28M (c.83A>T) (c) and H3-3A p.G35R (c.103G>A) (d) mutations in FFPE DNA. **Fig. S3** Detection of BRAF V600E (a) and BRAF V600K (b) mutations in FFPE DNA using ddPCR. **Fig. S4** Detection of H3-3A p.K28M and H3-3A p.G35R/V mutations in FFPE DNA using ddPCR. **Fig. S5** Detection of the PRKCA D463H mutation in chordoid glioma FFPE tissue samples using ddPCR. **Fig. S6** Analysis of copy number variations on chromosomal arms 1p and 19q in 15 glioma samples using ddPCR. **Fig. S7** Loss of heterozygosity (LOH) on chromosomal arms 1p and 19q (a, b) as well as on chromosome 10 (c) detected by ddPCR-based SNP analysis. **Fig. S8** Detection of *BRAF* duplication in 20 pilocytic astrocytomas by ddPCR analysis of *UBN2* and *BRAF* copy number using the assay reported by Appay et al. [8]. **Fig. S9** Job Report file of case 1 using the ARCHER® FusionPlex Panel and Archer Analysis software v6.2.7. **Fig. S10** The general workflow for the ddPCR-based molecular diagnostics

## References

[1] Louis DN, Perry A, Wesseling P (2021). The 2021 WHO classification of tumors of the central nervous system: a summary. Neuro Oncol.

[2] Louis DN, Wesseling P, Aldape K (2020). cIMPACT-NOW update 6: new entity and diagnostic principle recommendations of the cIMPACT-Utrecht meeting on future CNS tumor classification and grading. Brain Pathol.

[3] Brat DJ, Aldape K, Colman H (2020). cIMPACT-NOW update 5: recommended grading criteria and terminologies for IDH-mutant astrocytomas. Acta Neuropathol.

[4] Leske H, Dalgleish R, Lazar AJ (2021). A common classification framework for histone sequence alterations in tumours: an expert consensus proposal. J Pathol.

[5] Louis DN, Perry A, Reifenberger G (2016). The 2016 world health organization classification of tumors of the central nervous system: a summary. Acta Neuropathol.

[6] Pfister S, Janzarik WG, Remke M (2008). BRAF gene duplication constitutes a mechanism of MAPK pathway activation in low-grade astrocytomas. J Clin Invest.

[7] Jones DTW, Kocialkowski S, Liu L (2008). Tandem duplication producing a novel oncogenic BRAF fusion gene defines the majority of pilocytic astrocytomas. Cancer Res.

[8] Appay R, Fina F, Macagno N (2018). Duplications of KIAA1549 and BRAF screening by Droplet Digital PCR from formalin-fixed paraffin-embedded DNA is an accurate alternative for KIAA1549-BRAF fusion detection in pilocytic astrocytomas. Mod Pathol.

[9] Schindler G, Capper D, Meyer J (2011). Analysis of BRAF V600E mutation in 1,320 nervous system tumors reveals high mutation frequencies in pleomorphic xanthoastrocytoma, ganglioglioma and extra-cerebellar pilocytic astrocytoma. Acta Neuropathol.

[10] Rosenberg S, Simeonova I, Bielle F (2018). A recurrent point mutation in PRKCA is a hallmark of chordoid gliomas. Nat Commun.

[11] Goode B, Mondal G, Hyun M (2018). A recurrent kinase domain mutation in PRKCA defines chordoid glioma of the third ventricle. Nat Commun.

[12] Capper D, Zentgraf H, Balss J (2009). Monoclonal antibody specific for IDH1 R132H mutation. Acta Neuropathol.

[13] Bechet D, Gielen GGH, Korshunov A (2014). Specific detection of methionine 27 mutation in histone 3 variants (H3K27M) in fixed tissue from high-grade astrocytomas. Acta Neuropathol.

[14] Haque F, Varlet P, Puntonet J (2017). Evaluation of a novel antibody to define histone 3.3 G34R mutant brain tumours. Acta Neuropathol Commun.

[15] Yamamoto H, Iwasaki T, Yamada Y (2018). Diagnostic utility of histone H3.3 G34W, G34R, and G34V mutant-specific antibodies for giant cell tumors of bone. Hum Pathol.

[16] Reuss DE, Sahm F, Schrimpf D (2015). ATRX and IDH1-R132H immunohistochemistry with subsequent copy number analysis and IDH sequencing as a basis for an "integrated" diagnostic approach for adult astrocytoma, oligodendroglioma and glioblastoma. Acta Neuropathol.

[17] Capper D, Preusser M, Habel A (2011). Assessment of BRAF V600E mutation status by immunohistochemistry with a mutation-specific monoclonal antibody. Acta Neuropathol.

[18] Nikiforova MN, Wald AI, Melan MA (2016). Targeted next-generation sequencing panel (GlioSeq) provides comprehensive genetic profiling of central nervous system tumors. Neuro Oncol.

[19] Sahm F, Schrimpf D, Jones DTW (2016). Next-generation sequencing in routine brain tumor diagnostics enables an integrated diagnosis and identifies actionable targets. Acta Neuropathol.

[20] Zacher A, Kaulich K, Stepanow S (2017). Molecular diagnostics of gliomas using next generation sequencing of a glioma-tailored gene panel. Brain Pathol.

[21] Capper D, Jones DTW, Sill M (2018). DNA methylation-based classification of central nervous system tumours. Nature.

[22] Gorniak P, Ejduk A, Borg K (2016). Comparison of high-resolution melting analysis with direct sequencing for the detection of recurrent mutations in DNA methyltransferase 3A and isocitrate dehydrogenase 1 and 2 genes in acute myeloid leukemia patients. Eur J Haematol.

[23] Ball MK, Kollmeyer TM, Praska CE (2020). Frequency of false-positive FISH 1p/19q codeletion in adult diffuse astrocytic gliomas. Neurooncol Adv.

[24] Malzkorn B, Reifenberger G (2016). Practical implications of integrated glioma classification according to the World Health Organization classification of tumors of the central nervous system 2016. Curr Opin Oncol.

[25] Hindson BJ, Ness KD, Masquelier DA (2011). High-throughput droplet digital PCR system for absolute quantitation of DNA copy number. Anal Chem.

[26] Miotke L, Lau BT, Rumma RT (2014). High sensitivity detection and quantitation of DNA copy number and single nucleotide variants with single color droplet digital PCR. Anal Chem.

[27] Ichimura K, Schmidt EE, Goike HM (1996). Human glioblastomas with no alterations of the CDKN2A (p16INK4A, MTS1) and CDK4 genes have frequent mutations of the retinoblastoma gene. Oncogene.

[28] Corless B, Chang GA, Cooper S (2018). Development of novel mutation-specific droplet digital PCR assays detecting TERT promoter mutations in tumor and plasma samples. J Mol Diagn.

[29] Hirano M, Ohka F, Maeda S (2018). A novel high-sensitivity assay to detect a small fraction of mutant IDH1 using droplet digital PCR. Brain Tumor Pathol.

[30] Felsberg J, Hentschel B, Kaulich K (2017). Epidermal growth factor receptor variant III (EGFRvIII) positivity in EGFR-amplified glioblastomas: prognostic role and comparison between primary and recurrent tumors. Clin Cancer Res.

[31] Weller M, Kaulich K, Hentschel B (2014). Assessment and prognostic significance of the epidermal growth factor receptor vIII mutation in glioblastoma patients treated with concurrent and adjuvant temozolomide radiochemotherapy. Int J Cancer.

[32] Fontanilles M, Marguet F, Ruminy P (2020). Simultaneous detection of EGFR amplification and EGFRvIII variant using digital PCR-based method in glioblastoma. Acta Neuropathol Commun.

[33] Ishii N, Maier D, Merlo A (1999). Frequent co-alterations of TP53, p16/CDKN2A, p14ARF, PTEN tumor suppressor genes in human glioma cell lines. Brain Pathol.

[34] Armbruster DA, Pry T (2008). Limit of blank, limit of detection and limit of quantitation. Clin Biochem Rev.

[35] Weller M, Weber RG, Willscher E (2015). Molecular classification of diffuse cerebral WHO grade II/III gliomas using genome- and transcriptome-wide profiling improves stratification of prognostically distinct patient groups. Acta Neuropathol.

[36] Felsberg J, Wolter M, Seul H (2010). Rapid and sensitive assessment of the IDH1 and IDH2 mutation status in cerebral gliomas based on DNA pyrosequencing. Acta Neuropathol.

[37] Boström J, Cobbers JM, Wolter M (1998). Mutation of the PTEN (MMAC1) tumor suppressor gene in a subset of glioblastomas but not in meningiomas with loss of chromosome arm 10q. Cancer Res.

[38] Felsberg J, Erkwoh A, Sabel MC (2004). Oligodendroglial tumors: refinement of candidate regions on chromosome arm 1p and correlation of 1p/19q status with survival. Brain Pathol.

[39] Toedt G, Barbus S, Wolter M (2011). Molecular signatures classify astrocytic gliomas by IDH1 mutation status. Int J Cancer.

[40] Roerig P, Nessling M, Radlwimmer B (2005). Molecular classification of human gliomas using matrix-based comparative genomic hybridization. Int J Cancer.

[41] Tian Y, Rich BE, Vena N (2011). Detection of KIAA1549-BRAF fusion transcripts in formalin-fixed paraffin-embedded pediatric low-grade gliomas. J Mol Diagn.

[42] Lysiak M, Radke K, Malmström A (2017). P03.15 detection of 1p19q co-deletion in oligodendrogliomas with droplet digital PCR. Neuro-Oncology.

[43] Crespo I, Vital AL, Nieto AB (2011). Detailed characterization of alterations of chromosomes 7, 9, and 10 in glioblastomas as assessed by single-nucleotide polymorphism arrays. J Mol Diagn.

[44] Capper D, Stichel D, Sahm F (2018). Practical implementation of DNA methylation and copy-number-based CNS tumor diagnostics: the Heidelberg experience. Acta Neuropathol.

[45] Liu X-Y, Gerges N, Korshunov A (2012). Frequent ATRX mutations and loss of expression in adult diffuse astrocytic tumors carrying IDH1/IDH2 and TP53 mutations. Acta Neuropathol.

[46] Louis DN, Giannini C, Capper D (2018). cIMPACT-NOW update 2: diagnostic clarifications for diffuse midline glioma, H3 K27M-mutant and diffuse astrocytoma/anaplastic astrocytoma, IDH-mutant. Acta Neuropathol.

[47] Louis DN, Ellison DW, Brat DJ (2019). cIMPACT-NOW: a practical summary of diagnostic points from Round 1 updates. Brain Pathol.

[48] Wang J, Zhao Y, Li J (2015). IDH1 mutation detection by droplet digital PCR in glioma. Oncotarget.

[49] Ge J, Liu MY, Li L (2020). Detection of IDH1 and TERT promoter mutations with droplet digital PCR in diffuse gliomas. Int J Clin Exp Pathol.

[50] Muralidharan K, Yekula A, Small JL (2020). TERT promoter mutation analysis for blood-based diagnosis and monitoring of gliomas. Clin Cancer Res.

[51] Appay R, Fina F, Barets D (2020). Multiplexed droplet digital PCR assays for the simultaneous screening of major genetic alterations in tumors of the central nervous system. Front Oncol.

[52] Fina F, Barets D, Colin C (2017). Droplet digital PCR is a powerful technique to demonstrate frequent FGFR1 duplication in dysembryoplastic neuroepithelial tumors. Oncotarget.

[53] Martínez-Ricarte F, Mayor R, Martínez-Sáez E (2018). Molecular diagnosis of diffuse gliomas through sequencing of cell-free circulating tumor DNA from cerebrospinal fluid. Clin Cancer Res.

[54] Bruzek AK, Ravi K, Muruganand A (2020). Electronic DNA analysis of CSF cell-free tumor DNA to quantify multi-gene molecular response in pediatric high-grade glioma. Clin Cancer Res.

[55] Chen WW, Balaj L, Liau LM (2013). BEAMing and droplet digital PCR analysis of mutant IDH1 mRNA in glioma patient serum and cerebrospinal fluid extracellular vesicles. Mol Ther Nucleic Acids.

[56] McEvoy AC, Wood BA, Ardakani NM (2018). Droplet digital PCR for mutation detection in formalin-fixed, paraffin-embedded melanoma tissues: a comparison with sanger sequencing and pyrosequencing. J Mol Diagn.

[57] Rowlands V, Rutkowski AJ, Meuser E (2019). Optimisation of robust singleplex and multiplex droplet digital PCR assays for high confidence mutation detection in circulating tumour DNA. Sci Rep.

[58] Körber V, Yang J, Barah P (2019). Evolutionary trajectories of IDHWT glioblastomas reveal a common path of early tumorigenesis instigated years ahead of initial diagnosis. Cancer Cell.

[59] Decraene C, Bortolini Silveira A, Michel M (2018). Single droplet digital polymerase chain reaction for comprehensive and simultaneous detection of mutations in hotspot regions. JoVE.

[60] Brat DJ, Aldape K, Colman H (2018). cIMPACT-NOW update 3: recommended diagnostic criteria for “Diffuse astrocytic glioma, IDH-wildtype, with molecular features of glioblastoma, WHO grade IV”. Acta Neuropathol.

