# Supplementary material for: Droplet digital PCR-based analyses for robust, rapid, and sensitive molecular diagnostics of gliomas
Source: Acta Neuropathol Commun. 2022 Mar 31;10:42. doi: 10.1186/s40478-022-01335-6 (PMC8973808; doi:10.1186/s40478-022-01335-6)
Supplement: Supplementary file 1 — Additional file1. Table S1 Overview of the primer and probe sequences used for the individual ddPCR assays in this study including both previously published and newly designed assays. Table S2 Commercially available assays used for ddPCR-based single nucleotide variant (SNV) and copy number variation (CNV) analyses of certain biomarkers. Table S3 Primers and probes used for ddPCR-based single nucleotide polymorphism (SNP) analysis on chromosomal arms 1p and 19q. Table S4 Primers and probes used for ddPCR-based SNP analysis on chromosome 10. Table S5 Thermocycler conditions used for ddPCR assays. Table S6 Sensitivity, specificity, accuracy, and precision of each ddPCR assay investigated in relation to the respective method used for validation. Table S7 Detection of IDH1 and IDH2 mutations using duplex and multiplex ddPCR assays. Table S8 Detection of BRAF V600E and BRAF V600K mutations using duplex ddPCR assays. Table S9 Detection of H3-3A p.K28M and H3-3A p.G35R mutations using duplex ddPCR assays. Table S10 Comparison of the experimentally detected mean allele frequency (AF) of deleted SNP loci on 1p and 19q versus the AF calculated from the mutant allele frequency (MAF) of TERTp (a) or IDH1/2 (b) mutations. Table S11 Comparison of the experimentally detected mean allele frequency (AF) of deleted SNP loci on chromosome 10 versus the AF calculated from the mutant allele frequency (MAF) of TERTp mutations. Table S12 Detection of the EGFRvIII variant using ddPCR as well as comparison of a commercially available PrimePCR™ ddPCR (a) and a self-designed (b) copy number assay for EGFR exon 28. Table S13 Determination of CDKN2A copy number in 66 glioma samples using a PrimePCR™ ddPCR CDKN2A copy number assay. Fig. S1 Detection of IDH1 and IDH2 hotspot mutations in FFPE DNA using ddPCR. Fig. S2 Sensitivity of ddPCR to detect IDH1 R132H (a), BRAF V600E (b) as well as H3-3A p.K28M (c.83A>T) (c) and H3-3A p.G35R (c.103G>A) (d) mutations in FFPE DNA. Fig. S3 Detection of BRAF V [file 40478_2022_1335_MOESM1_ESM.pdf]

Droplet digital PCR-based analyses for robust, rapid, and sensitive molecular diagnostics of gliomas

Marietta Wolter[1], Jörg Felsberg[1], Bastian Malzkorn[1], Kerstin Kaulich[1], Guido Reifenberger[1,2]

[1]Institute of Neuropathology, Heinrich Heine University, Medical Faculty, and University Hospital Düsseldorf, Düsseldorf, Germany

[2]German Cancer Consortium (DKTK), German Cancer Research Center (DKFZ) Heidelberg, partner site Essen/Düsseldorf, Germany

SUPPLEMENTARY INFORMATION

Supplementary Tables

Supplementary Table 1: Overview of the primer and probe sequences used for the individual ddPCR assays in this study including both previously published and newly designed assays. Sequences of primers and probes used for single nucleotide variant (SNV) and copy number variation (CNV) analyses are shown together with the chromosomal location of the investigated genes, fluorescent labeling at the 5´- and 3´-ends of the probes and amplicon length as well as the source of the individual primer/probe sequences.

Gene	Locus	Application	Forward Primer 5´-3´	Reverse Primer 5´-3´	Probe 5´-3´	Amplicon size in base pair	target or reference	Source
RPPH1	14q11.2	CNV	GAGCTTGGAACAGACTCAC	AGAGTAGTCTGAATTGGGTTATG	HEX-AGCGAAGTGAGTTCAATGGCTGAGGT-BHQ1	93	reference	https://eu.idtdna.com/PrimerQuest/
NCKAP5	2q21.2	CNV	CATGACTGCGAGCCCAAGATG	TGCTCTGTTCTCAGGCAGAC	HEX-CTAGAAATGTTGGCCATTATTTTGCTACCCTGTACT-BHQ1	86	reference	self-designed
NCKAP5	2q21.2	CNV	GTAATGCAGGTTTGGCCATT	ATGGGCTCATCTCTGAAAC	HEX-TGCATTCCAACCCCATGGCCTCAGT-BHQ1	71	reference	self-designed
EIF4E3	3p13	CNV	CAGAGCTGGGTATTGAAGTC	CTTGATTAGGGTGGCTAGTG	HEX-AGTTGTATCCCGGTGCAGGCGT-BHQ1	109	reference	https://eu.idtdna.com/PrimerQuest/
TMEM213	7q34	CNV	CAACAGCAAAGTGGAAGAAAGGAT	CTCCTGGTCATGTCCCAGTTC	HEX-CCGCATTCTACAGGATCGGCCTTAGC-BHQ1	101	reference	Ref. [1]
GBAS	7p11.2	CNV	CGTGACAACACCTTTGTTATTC	CTTGGATCAACTTTCCGGACAA	HEX-AGGACATGGACATCTTCCAGCAACAGA-BHQ1	105	reference	https://eu.idtdna.com/PrimerQuest/
SDK1	7p22.2	CNV; chr7	CCACAGATCCACCTGGAAG	TGAGCTCACTGTCATCGC	6-FAM-ACCGCCTTGTTCTCACCTGCCT-BHQ1	100	target	https://eu.idtdna.com/PrimerQuest/
STK31	7p15.3	CNV; chr7	GGTGGTCTCCTTACAATG	CCTCAGAACGTACCAAAG	6-FAM-ACGCTTGCTGCTAAGTTCCTTCA-BHQ1	94	target	http://www.oligoarchitect.com
GLI3	7p14.1	CNV; chr7	ACTTGGAGGGCGTGTTA	TAGTCTCGAGTAGGCCTTTG	6-FAM-AAGCTGCTGTTGCCATTGTGCAGA-BHQ1	107	target	https://eu.idtdna.com/PrimerQuest/
GBAS	7p11.2	CNV; chr7	CGTGACAACACCTTTGTTATTC	CTTGGATCAACTTTCCGGACAA	6-FAM-AGGACATGGACATCTTCCAGCAACAGA-BHQ1	105	target	https://eu.idtdna.com/PrimerQuest/
PDK4	7q21.3	CNV; chr7	GTGGCTTTGATGACTTGTTTG	TGGTGCAGTGGAGTATGT	6-FAM-TCAGACAGAGGAGGTGGTGTTCCC-BHQ1	106	target	https://eu.idtdna.com/PrimerQuest/
KCND2	7q31.31	CNV; chr7	GTGACTACAGCAATAATAAGC	CTGACAATATTTCCTCCTGA	6-FAM-AGTAACCACACCAGAAGGAGACGA-BHQ1	101	target	http://www.oligoarchitect.com
TMEM213	7q34	CNV; chr7	CAACAGCAAAGTGGAAGAAAGGAT	CTCCTGGTCATGTCCCAGTTC	6-FAM-CCGCATTCTACAGGATCGGCCTTAGC-BHQ1	101	target	Ref. [1]
SHH	7q36.3	CNV; chr7	CGAGTCCAAGGCACATATC	TTGAATCAAGCCGAGGTG	6-FAM-ATCCGACCCAAGGAAGGCCATTGG-BHQ1	105	target	https://eu.idtdna.com/PrimerQuest/
BRAF	7q34	CNV; BRAF duplication	CAAGTCACCACAAAAACCTATCGT	ATACAAAGAAACAGCAAAATGGTGAT	HEX-AGAGTCTTCCTGCCCAACAAACAGAGGAC-BHQ1	100	reference	Ref. [1]
BRAF	7q34	CNV; BRAF duplication	AAATGATGTTTTGTTTTTCAGTTACTT	GCAGACAAATTTCAGGAAGGA	6-FAM-ACGCCAAGTCAATCATCCACAGAGA-BHQ1	92	target	Ref. [1]
UBN2	7q34	CNV; BRAF duplication	GGAGCTCAGCATGCAGCAA	GATGAAGGAGGTGGCGTTCTC	6-FAM-ACTCACCTCTGCCTGCACACTTACAGCAAG-BHQ1	90	target	Ref. [1]
EGFR	7p11.2	CNV; EGFR	CGAAGGAAAGTAAGGAGCTG	AAGAAGATGCACGAAGGC	6-FAM-AGCTCACATTCATAGGTGCCGCCABHQ1	64	target	https://eu.idtdna.com/PrimerQuest/
TP73	1p36.32	CNV; LOH 1p	CTCATCCGCGTGGAAGGCAA	CCAAAGATCCCACCTGAGCAT	6-FAM-CCAAGACTGGCCAGCGGCTTCC-BHQ1	70	target	PrimerExpress 3.0.1 (ThermoFisher Scientific)
CDKN2C	1p32.3	CNV; LOH 1p	TACGCTGCGGAGCCTCAAAG	GGCGAGGACAGGGGTTTG	6-FAM-CCCTGCGGCCCTCCACAACCGT-BHQ1	68	target	www.genscript.com/tools/real-time-pcr-tagman-primer-design-tool
FUBP1	1p31.1	CNV; LOH 1p	CTCCTACCCTCACTGTCACAT	GCAATTTTGGCCGTTTCCT	6-FAM-CCCTGCCAGCTCCTGCACCC-BHQ1	68	target	PrimerExpress 3.0.1 (ThermoFisher Scientific)
LRIG2	1p13.2	CNV; LOH 1p	CCAGACTGTTCTGCTTCTTC	GTTCCGAGTCCTACTAAAATC	6-FAM-CCTGCCACAGGTTACAGGATCATGCT-BHQ1	75	target	https://eu.idtdna.com/PrimerQuest/
CCNE1	19q12	CNV; LOH 19q	CCCACACCTGACAAAGAAG	CCTCTGGATGGTGCAATAAT	6-FAM-TACCCAAACTCAACGTGCAAGCCTCG-BHQ1	80	target	https://eu.idtdna.com/PrimerQuest/
CEBPA	19q13.11	CNV; LOH 19q	GAGCCAGGACTAGGAGATT	AGAGGAAGGGAGGGGAC	6-FAM-TCCTGAAAGCCTGGCCTGCT-BHQ1	70	target	https://eu.idtdna.com/PrimerQuest/
ERCC1	19q13.32	CNV; LOH 19q	CAGTACCCCGAGAGAAAGC	GGGGCTTCTGAGCCTGAC	6-FAM-TTCTCACCAGGCTCAACAAGGGCC-BHQ1	86	target	https://eu.idtdna.com/PrimerQuest/
NLRP9	19q13.42	CNV; LOH 19q	TTGCAGGCCAGGGCTGT	CAGTTGGTATCAGGACGCCAT	6-FAM-TATGTGGGCCAGCGATGAACTGGG-BHQ1	70	target	https://eu.idtdna.com/PrimerQuest/
BRAF	7q34	SNV; V600E	CTACTGTTTTCCTTTACTTACTACACCTCAGA	ATCCAGACAACTGTTCAAACTGATG	6-FAM-TAGCTACAGAGAAATC-MGBEQ	136	target	Ref. [2]
BRAF	7q34	SNV; V600K	CTACTGTTTTCCTTTACTTACTACACCTCAGA	ATCCAGACAACTGTTCAAACTGATG	6-FAM-CTAGCTACAAAGAAATCT-MGBEQ	136	target	primer according to Ref. [1], probe self-designed
BRAF	7q34	SNV; Wildtype V600	CTACTGTTTTCCTTTACTTACTACACCTCAGA	ATCCAGACAACTGTTCAAACTGATG	HEX-CTAGCTACAGTGAAATC-MGBEQ	136	reference	Ref. [2]
IDH1	2q34	SNV; Wildtype R132	CTTGTGAGTGGATGGGTAAAACCTA	CCAACATGACTTACTTGATCCCCATA	HEX-CATCATAGGTCGTCATGC-MGBEQ	71	reference	Ref. [3]
IDH1	2q34	SNV; R132H	CTTGTGAGTGGATGGGTAAAACCTA	CCAACATGACTTACTTGATCCCCATA	6-FAM-ATCATAGGTCATCATGC-MGBEQ	71	target	Ref. [3]
IDH1	2q34	SNV; R132L	CTTGTGAGTGGATGGGTAAAACCTA	CCAACATGACTTACTTGATCCCCATA	6-FAM-ATCATAGGTCTTCATGC-MGBEQ	71	target	modified according to Ref. [3]
IDH1	2q34	SNV; R132S	CTTGTGAGTGGATGGGTAAAACCTA	CCAACATGACTTACTTGATCCCCATA	6-FAM-ATCATAGGTAGTCATGC-MGBEQ	71	target	modified according to Ref. [3]
IDH1	2q34	SNV; R132C	CTTGTGAGTGGATGGGTAAAACCTA	CCAACATGACTTACTTGATCCCCATA	6-FAM-ATCATAGGTTGTCATGC-MGBEQ	71	target	modified according to Ref. [3]
IDH1	2q34	SNV; R132G	CTTGTGAGTGGATGGGTAAAACCTA	CCAACATGACTTACTTGATCCCCATA	6-FAM-ATCATAGGTGGTCATGC-MGBEQ	71	target	modified according to Ref. [3]
IDH2	15q26.1	SNV; Wildtyp R172	ACGCCTAGTCCCTGGCTG	GTGCCCAGGTCAGTGGATC	HEX-CCATTGGCAGGCACGC-MGBEQ	100	reference	self-designed
IDH2	15q26.1	SNV; R172G	ACGCCTAGTCCCTGGCTG	GTGCCCAGGTCAGTGGATC	6-FAM-CATTGGCGGGCACGC-MGBEQ	100	target	self-designed
IDH2	15q26.1	SNV; R172W	ACGCCTAGTCCCTGGCTG	GTGCCCAGGTCAGTGGATC	6-FAM-CATTGGCTGGCACGC-MGBEQ	100	target	self-designed
IDH2	15q26.1	SNV; R172K	ACGCCTAGTCCCTGGCTG	GTGCCCAGGTCAGTGGATC	6-FAM-CATTGGCAAGCACGC-MGBEQ	100	target	self-designed
IDH2	15q26.1	SNV; R172M	ACGCCTAGTCCCTGGCTG	GTGCCCAGGTCAGTGGATC	6-FAM-CATTGGCATGCACGC-MGBEQ	100	target	self-designed
IDH2	15q26.1	SNV; R172S	ACGCCTAGTCCCTGGCTG	GTGCCCAGGTCAGTGGATC	6-FAM-CATTGGCAGTCACGC-MGBEQ	100	target	self-designed
H3F3A	1q42.12	SNV; Wildtyp G35	GCAACTGGCTACAAAAGC	GTGGATACATACAAGAGAGAC	HEX-TACTGGAGGGGTGAA-MGBEQ	124	reference	http://www.oligoarchitect.com
H3F3A	1q42.12	SNV; G35R (c.103,G>A)	GCAACTGGCTACAAAAGC	GTGGATACATACAAGAGAGAC	6-FAM-CTACTGGAAGGGTGAA-MGBEQ	124	target	modified according to oligoarchitect (http://www.oligoarchitect.com)
H3F3A	1q42.12	SNV; G35R (c.103,G>C)	GCAACTGGCTACAAAAGC	GTGGATACATACAAGAGAGAC	6-FAM-TACTGGACGGGTGAA-MGBEQ	124	target	modified according to oligoarchitect (http://www.oligoarchitect.com)
H3F3A	1q42.12	SNV; G35V (c.104, G>T)	GCAACTGGCTACAAAAGC	GTGGATACATACAAGAGAGAC	6-FAM-CTACTGGAGTGGTGAA-MGBEQ	124	target	modified according to oligoarchitect (http://www.oligoarchitect.com)
H3F3A	1q42.12	SNV; G35W (c.103, G>T)	GCAACTGGCTACAAAAGC	GTGGATACATACAAGAGAGAC	6-FAM-CTACTGGATGGGTGAA-MGBEQ	124	target	modified according to oligoarchitect (http://www.oligoarchitect.com)
PRKCA	17q24.2	SNV; Wildtyp D463	TCATGGGAAGGACTCTGATGTTAA	TATGTCCTTCTGAATCCAACATGAC	HEX-CCTTGCAGG**G**ATCTGAAG-MGBEQ	83	reference	self-designed primers, Probe by OligoArchitekt (http://www.oligoarchitect.com)
PRKCA	17q24.2	SNV; D463H	TCATGGGAAGGACTCTGATGTTAA	TATGTCCTTCTGAATCCAACATGAC	6-FAM-CCTTGCAGG**C**ATCTGAAG-MGBEQ	83	target	self-designed primers, Probe by OligoArchitekt (http://www.oligoarchitect.com)

Supplementary Table 2: Commercially available assays used for ddPCR-based single nucleotide variant (SNV) and copy number variation (CNV) analyses of certain biomarkers.

Gene	Locus	Application	Location Amplicon	RefSeq	Amplicon size in base pair	Assay ID	5´-Dye	3´-Quencher	Assay name	Company
TERT	5p15.33	SNV; "C228T"	Promoter	NC_000005.9	113	dHsaEXD72405942	6-FAM mut/HEX wt	Iowa Black FQ	ddPCR Expert Design Assay	Bio-Rad Laboratories, Munic, Germany
TERT	5p15.33	SNV; "C250T"	Promoter	NC_000005.9	113	dHsaEXD46675715	6-FAM mut/HEX wt	Iowa Black FQ	ddPCR Expert Design Assay	Bio-Rad Laboratories, Munic, Germany
H3F3A	1q42.12	SNV; K28M	Exon 2	NM_002107	129	dHsaCP2500510	6-FAM	Iowa Black FQ	PrimePCR™ ddPCR Mutation Assay	Bio-Rad Laboratories, Munic, Germany
H3F3A	1q42.12	SNV; Wildtype K28	Exon 2	NM_002107	129	dHsaCP2500511	HEX	Iowa Black FQ	PrimePCR™ ddPCR Mutation Assay	Bio-Rad Laboratories, Munic, Germany
CDKN2A	9p21.3	CNV	Intron 1	NM_058195.3	66	dHsaCP1000581	6-FAM	Iowa Black FQ	PrimePCR™ ddPCR Copy Number Assay	Bio-Rad Laboratories, Munic, Germany
EGFR	7p11.2	CNV	Exon 7	NM_005228	64	dHsaCP2500318	6-FAM	Iowa Black FQ	PrimePCR™ ddPCR Copy Number Assay	Bio-Rad Laboratories, Munic, Germany
EGFR	7p11.2	CNV	Exon 28	NM_005228	88	dHsaCP1000020	6-FAM	Iowa Black FQ	PrimePCR™ ddPCR Copy Number Assay	Bio-Rad Laboratories, Munic, Germany

Supplementary Table 3: Primers and probes used for ddPCR-based single nucleotide polymorphism (SNP) analysis on chromosomal arms 1p and 19q.

a) Primers und probes used for initial ddPCR-based analysis of 5 SNPs on 1p and on 19q.

	SNP*	Locus	Reference Allele*	Observed Alleles*	Average Heterozygosity*	Allele Frequency [%]*	Allele Frequency [%]*	Forward Primer 5´-3´	Reverse Primer 5´-3´	5´-Fluoro-phore	Probe 5´-3´	3´-Quencher	5´-Fluoro-phore	Probe 5´-3´	3´-Quencher	Amplicon size in base pair
chr 1p	rs4648379	1p36.32	C	C/T	0.466 +/-0.126	C: 63.00	T: 37.00	CTTGTTTTCTGTGGGGCCCT	AGACATGATCCTCCTGTGTGC	6-FAM	CCTCCCTTATGCATGA	MGB-Eclipse	HEX	CCTCCCTCATGCATG	MGB-Eclipse	91
	rs3157	1p34.2	A	A/G	0.467 +/-0.124	A: 62.82	G: 37.18	CGGCCTGATTTTCTTCGACAG	TAGGCCTCATCTGTGGGAAG	6-FAM	TCCCTGGCTCATACT	MGB-Eclipse	HEX	TCCCTGGCTTATACTG	MGB-Eclipse	95
	rs9787003	1p31.1	G	A/G	0.475 +/-0.109	A: 38.78	G: 61.22	CTCTTCCCAAATTTTTCTCTTTTAAGAG	GAGCTCAAGGCTGCAGTGAG	6-FAM	TCTCTGTCACCCAAACT	MGB-Eclipse	HEX	CTCTGTCGCCCAAAC	MGB-Eclipse	94
	rs9428240	1p12	C	C/T	0.497 +/-0.041	C: 54.11	T: 45.89	CAGCATTTAGGATTTTATATTCTGGG	CATCGAGACATCCCTTATCCT	6-FAM	AATGAGTGTCTTTGTCAC	MGB-Eclipse	HEX	AATGAGTGCCTTTGTCA	MGB-Eclipse	97
	rs1493695	1p12	T	C/T	0.469 +/-0.121	C: 37.52	T: 62.48	CAGTCCTTTTTCCATCTCCTAGTA	CAGCCTGAGGCACCCATAC	6-FAM	TGGACTTTCCTTGTCTT	MGB-Eclipse	HEX	TGGACTTTCTTTGTCTTC	MGB-Eclipse	91

	SNP*	Locus	Reference Allele*	Observed Alleles*	Average Heterozygosity*	Allele Frequency [%]*	Allele Frequency [%]*	Forward Primer 5´-3´	Reverse Primer 5´-3´	5´-Fluoro-phore	Probe 5´-3´	3´-Quencher	5´-Fluoro-phore	Probe 5´-3´	3´-Quencher	Amplicon size in base pair
chr 19q	rs4805965	19q12	A	A/G	0.443 +/-0.159	A: 33.04	G: 66.95	CTAGTTTTGTGAAAAATGATTTATTTCAAAAC	CACTTTTCTCTCCTGTGGCCT	6-FAM	CCATGGCAAAAGGGG	MGB-Eclipse	HEX	CCATGGCGAAAGGG	MGB-Eclipse	97
	rs6508980	19q13.2	A	A/G	0.468 +/-0.123	A: 37.30	G: 62.70	CACACCCTTTTCAATAATTGGTAC	GTAAACATGGTAAGAAATAGGAGTTC	6-FAM	AGTTGCATTATCATATGC	MGB-Eclipse	HEX	AGTTGCATTGTCATATG	MGB-Eclipse	88
	rs10404903	19q13.32	T	C/T	0.478 +/-0.102	C: 60.46	T: 39.54	TGGGCCAGTCTGGACTGG	CTAAGTCCCCCCTCTAGACC	6-FAM	AATTTGGGTGTGATCAAA	MGB-Eclipse	HEX	AATTTGGGCGTGATCAA	MGB-Eclipse	97
	rs10424927	19q13.43	C	C/G/T	0.476 +/-0.107	C: 61.00	T: 39.00	GTTTGCCCTGGGAATACTGC	GTGTGAACCACCGTGCC	6-FAM	CAGTGAGTTGCACTTT	MGB-Eclipse	HEX	CAGTGAGCTGCACTT	MGB-Eclipse	100
	rs260464	19q13.43	G	G/T	0.499 +/-0.026	G: 47.40	T: 52.60	GTCCTAGCTACTCAGGAGG	AGCGGTGTGATGGCATGATCA	6-FAM	CTGCAACCACCGCCT	MGB-Eclipse	HEX	CTGCAACAACCGCCTC	MGB-Eclipse	91

b) Primers und probes used for ddPCR-based analysis of 4 additional SNPs on 1p and on 19q in cases with only one or without any informative SNP per chromosomal arm.

	SNP*	Locus	Reference Allele*	Observed Alleles*	Average Heterozygosity*	Allele Frequency [%]*	Allele Frequency [%]*	Forward Primer 5´-3´	Reverse Primer 5´-3´	5´-Fluoro-phore	Probe 5´-3´	3´-Quencher	5´-Fluoro-phore	Probe 5´-3´	3´-Quencher	Amplicon size in base pair
chr 1p	rs2281168	1p36.21	C	C/T	0.471 +/- 0.117	C: 62.10	T: 37.90	TTGCCCCCTCCATAGGATG	CTGGGGCTGGAGAGGAG	6-FAM	TCCTCGCTTACGTCGAT	MGB-Eclipse	HEX	TCCTCGCTCACGTCGA	MGB-Eclipse	94
	rs2165194	1p33	A	A/G	0.443 +/- 0.159	A: 66.95	G: 33.05	GTCTCTTTGTGCCCTCTGC	GTGCGCTCCAAGCAGGAAG	6-FAM	TGCACTCTGTGGACAT	MGB-Eclipse	HEX	TGCACTCTATGGACATA	MGB-Eclipse	95
	rs4551638	1p21.3	G	G/C	0.487 +/- 0.080	G: 58.07	C: 41.93	AGTTTGTTCAGGGAAACTCCTG	TGAATCATGGGGGCAGGTCATT	6-FAM	TCTCATGACATCTCACT	MGB-Eclipse	HEX	TCTCATGAGATCTCACT	MGB-Eclipse	97
	rs10776720	1p13.2	T	A/C/T	0.496 +/- 0.045	T: 54.51	A: 45.49	CCTCTTCTTTCAGATTGAAGAACTC	GAAGACTTTCTCACATAAATAAAAGCTG	6-FAM	AGGACAATACTGGTGCT	MGB-Eclipse	HEX	AGGACAATTCTGGTGCT	MGB-Eclipse	99

	SNP*	Locus	Reference Allele*	Observed Alleles*	Average Heterozygosity*	Allele Frequency [%]*	Allele Frequency [%]*	Forward Primer 5´-3´	Reverse Primer 5´-3´	5´-Fluoro-phore	Probe 5´-3´	3´-Quencher	5´-Fluoro-phore	Probe 5´-3´	3´-Quencher	Amplicon size in base pair
chr 19q	rs4422091	19q13.11	T	C/T	0.453 +/- 0.146	C: 65.40	T: 34.60	GTTTATTCTGGGTTTCTGCCCC	AGGTCACAGGGCAAACTGTTG	6-FAM	TCTGTCCATCTGTCAGT	MGB-Eclipse	HEX	CTGTCCACCTGTCAG	MGB-Eclipse	93
	rs7250409	19q13.13	C	C/T	0.482 +/- 0.094	C: 59.57	T: 40.44	GACGTGATTTGGATTCAGGCAG	GTCTTGCTCGGGTTTGAAAACC	6-FAM	AGAGTTAAAATACAGCTC	MGB-Eclipse	HEX	AGAGTTAAGATACAGCT	MGB-Eclipse	95
	rs830146	19q13.33	C	C/T	0.492 +/- 0.061	C: 43.85	T: 56.15	CAGAATGCTTTGCAGTCTCCC	AGCCTCTTCTACTTCCTGCCT	6-FAM	TGTAGCGTGCCTGGG	MGB-Eclipse	HEX	TTGTAGCGCGCCTGG	MGB-Eclipse	94
	rs12463376	19q13.41	G	G/A	0.497 +/- 0.040	G: 45.99	A: 54.01	CAGCAAGAGGGAGTATAAAGAGA	TTCACCCCAAGGCTCTGGT	6-FAM	TCTCTTGCCTGGACTG	MGB-Eclipse	HEX	TCTCTTGCTTGGACTGC	MGB-Eclipse	90

*, UCSC Genome Browser https://genome.euro.ucsc.edu/ (GRCh37/hg19)

Supplementary Table 4: Primers and probes used for ddPCR-based SNP analysis on chromosome 10.

	SNP*	Locus	Reference Allele*	Observed Alleles*	Average Heterozygosity*	Allele Frequency [%]*	Allele Frequency [%]*	Forward Primer 5´-3´	Reverse Primer 5´-3´	5´-Fluoro-phore	Probe 5´-3´	3´-Quencher	5´-Fluoro-phore	Probe 5´-3´	3´-Quencher	Amplicon size in base pair
chr10p	rs1668538	10p15.2	C	C/T	0.491 +/- 0.066	T: 43.37	C: 56.63	ATTCTTTCTAAAGGCTTTGTGAAG[illegible]	CTTTTGCTTTCTGGAAACCTTATTAC	6-FAM	CTGTAAAACACGATTGCC	MGB-Eclipse	HEX	TGTAAAACGCGATTGCC	MGB-Eclipse	97
	rs10904642	10p13	G	A/G	0.489 +/- 0.075	A: 42.45	G: 57.55	CTGTGTGTTAGGGTTTGCTTTTTA[illegible]	AGAAGCAATGTGGGTCACAGC	6-FAM	ATGGCCAACATCTGTG	MGB-Eclipse	HEX	ATGGCCAGCATCTGT	MGB-Eclipse	94
	rs7071770	10p12.1	T	C/T	0.496 +/- 0.047	C: 45.33	T: 54.67	TATGTGGCCTTTATTATGTTCCAG	TAACAGTAGAGACCTCACAGG	6-FAM	TTTGAAGTGCTTCACATA	MGB-Eclipse	HEX	TTGAAGTGTTTCACATAC	MGB-Eclipse	96
	rs660620	10p11.21	C	C/T	0.500 +/- 0.014	C: 48.60	T: 51.40	CCTAGGAAGTTAAGTCTTGGGGA	GTTTTCTCCCACGCCTTGTG	6-FAM	TGTGTTCCCGGCCACT	MGB-Eclipse	HEX	TGTGTTCCTGGCCACTC	MGB-Eclipse	94

	SNP*	Locus	Reference Allele*	Observed Alleles*	Average Heterozygosity*	Allele Frequency [%]*	Allele Frequency [%]*	Forward Primer 5´-3´	Reverse Primer 5´-3´	5´-Fluoro-phore	Probe 5´-3´	3´-Quencher	5´-Fluoro-phore	Probe 5´-3´	3´-Quencher	Amplicon size in base pair
chr10q	rs1343043	10q21.1	G	A/G/T	0.448 +/- 0.152	A: 33.95	G: 66.05	ATGTCTTTAGCCCTCTCTGTC	GAAAGACAAACATTATAACTGGAATTAT[illegible]	6-FAM	TGCTTTATATACTCTTCT	MGB-Eclipse	HEX	TGCTTTATGTACTCTTC	MGB-Eclipse	95
	rs4934493	10q23.31	G	A/G	0.498 +/- 0.032	A: 46.77	G: 53.24	TCTCCATATCCTGTGCTCACT	GATAACTTCTGCTTTCCTTGTGC	6-FAM	TTTTTTCTAAGCGAGACTC	MGB-Eclipse	HEX	TTTTTCTAGGCGAGACTC	MGB-Eclipse	95
	rs626989	10q25.1	C	C/G	0.500 +/- 0.000	C: 49.98	G: 50.02	GAAGTTGCTTTGGGTGAATCAG	GTGTACCATGTTTATAAAGTCTATAGTA[illegible]	6-FAM	TGGTGAGTCAATGCAAA	MGB-Eclipse	HEX	TGGTGAGTGAATGCAAA	MGB-Eclipse	97
	rs11816275	10q26.2	T	C/T	0.477 +/- 0.104	C: 39.36	T: 60.64	GCATCCAAGGACTCTCAGTAAC	AAGAAGGGTGTAAGGGAGGCAA	6-FAM	CTAAGTGGCATTTATCT	MGB-Eclipse	HEX	ACTAAGTGGTATTTATCTA	MGB-Eclipse	88

*, UCSC Genome Browser https://genome.euro.ucsc.edu/ (GRCh37/hg19)

Supplementary Table 5: Thermocycler conditions used for ddPCR assays.

PCR conditions for CNV assays:

CDKN2A, EGFR

Cycling Step	Temperature	Time	Ramp Rate	Number of Cycles
Enzyme activation	95°C	10 min		1
Denaturation	94°C	30 s	2.0°C/s	40
Annealing/Extension	60°C	1 min		
Enzyme deactivation	98°C	10 min		1
Hold (optional)	12°C	infinite	1.0°C/s	1

PCR conditions for SNV HotSpot assays:

TERT

Cycling Step	Temperature	Time	Ramp Rate	Number of Cycles
Enzyme activation	95°C	10 min		1
Denaturation	96°C	30 s	2.5°C/s	50
Annealing/Extension	62°C	1 min		
Enzyme deactivation	98°C	10 min	1.0°C/s	1
Hold (optional)	12°C	infinite	2.0°C/s	1

According to the manufacturer´s instruction a final concentration of 0.5 M Betaine and 1 mM EDTA was added to the 20 µl PCR mixture.

IDH1 and IDH2

Cycling Step	Temperature	Time	Ramp Rate	Number of Cycles
Enzyme activation	95°C	10 min		1
Denaturation	94°C	30 s	2.0°C/s	40
Annealing/Extension	53°C	1 min		
Enzyme deactivation	98°C	10 min		1
Hold (optional)	12°C	infinite	1.0°C/s	1

BRAF

Cycling Step	Temperature	Time	Ramp Rate	Number of Cycles
Enzyme activation	95°C	10 min		1
Denaturation	96°C	30 s	2.5°C/s	50
Annealing/Extension	62°C	1 min		
Enzyme deactivation	98°C	10 min	1.0°C/s	1
Hold (optional)	12°C	infinite	2.0°C/s	1

H3-3A

Cycling Step	Temperature	Time	Ramp Rate	Number of Cycles
Enzyme activation	95°C	10 min		1
Denaturation	94°C	30 s	2.0°C/s	40
Annealing/Extension	55°C	1 min		
Enzyme deactivation	98°C	10 min		1
Hold (optional)	12°C	infinite	1.0°C/s	1

PRKCA

Cycling Step	Temperature	Time	Ramp Rate	Number of Cycles
Enzyme activation	95°C	10 min		1
Denaturation	94°C	30 s	2.0°C/s	40
Annealing/Extension	60°C	1 min		
Enzyme deactivation	98°C	10 min		1
Hold (optional)	12°C	infinite	1.0°C/s	1

PCR conditions for SNP assays:

chr1p
rs4648379, rs3157, rs9787003, rs1493695, rs9428240

Cycling Step	Temperature	Time	Ramp Rate	Number of Cycles
Enzyme activation	95°C	10 min		1
Denaturation	94°C	30 s	2.0°C/s	50
Annealing/Extension	55.8°C	1 min		
Enzyme deactivation	98°C	10 min		1
Hold (optional)	12°C	infinite	1.0°C/s	1

chr19q
rs4805965, rs6508980, rs10404903

Cycling Step	Temperature	Time	Ramp Rate	Number of Cycles
Enzyme activation	95°C	10 min		1
Denaturation	94°C	30 s	2.0°C/s	50
Annealing/Extension	55.8°C	1 min		
Enzyme deactivation	98°C	10 min		1
Hold (optional)	12°C	infinite	1.0°C/s	1

chr19q
rs10424927, rs260464

Cycling Step	Temperature	Time	Ramp Rate	Number of Cycles
Enzyme activation	95°C	10 min		1
Denaturation	94°C	30 s	2.0°C/s	40
Annealing/Extension	60°C	1 min		
Enzyme deactivation	98°C	10 min		1
Hold (optional)	12°C	infinite	1.0°C/s	1

chr10p
rs1668538, rs10904642, rs7071770, rs1660620

Cycling Step	Temperature	Time	Ramp Rate	Number of Cycles
Enzyme activation	95°C	10 min		1
Denaturation	94°C	30 s	2.0°C/s	40
Annealing/Extension	60°C	1 min		
Enzyme deactivation	98°C	10 min		1
Hold (optional)	12°C	infinite	1.0°C/s	1

chr10q
rs4934493, rs626989, rs11816275

Cycling Step	Temperature	Time	Ramp Rate	Number of Cycles
Enzyme activation	95°C	10 min		1
Denaturation	94°C	30 s	2.0°C/s	40
Annealing/Extension	60°C	1 min		
Enzyme deactivation	98°C	10 min		1
Hold (optional)	12°C	infinite	1.0°C/s	1

chr10q
rs1343043

Cycling Step	Temperature	Time	Ramp Rate	Number of Cycles
Enzyme activation	95°C	10 min		1
Denaturation	94°C	30 s	2.0°C/s	40
Annealing/Extension	53°C	1 min		
Enzyme deactivation	98°C	10 min		1
Hold (optional)	12°C	infinite	1.0°C/s	1

chr7

Cycling Step	Temperature	Time	Ramp Rate	Number of Cycles
Enzyme activation	95°C	10 min		1
Denaturation	94°C	30 s	2.0°C/s	50
Annealing/Extension	55°C	1 min		
Enzyme deactivation	98°C	10 min		1
Hold (optional)	12°C	infinite	1.0°C/s	1

CNV, copy number variation; *SNV*, single nucleotide variant; *SNP*, single nucleotide polymorphism

Supplementary Table 6: Sensitivity, specificity, accuracy, and precision of each ddPCR assay investigated in relation to the respective method used for validation.

	TERTp mutation	IDH mutation	BRAF mutation	H3-3A mutation	PRKCA mutation	1p/19q co-deletion	deletion on chr10	gain on chr7	EGFR amplifi-cation	EGFRvIII deletion	BRAF dupli-cation	CDKN2A deletion
Sensitivity (= TP/(TP+FN))	100%	100%	100%	100%	100%	100%	88%	100%	100%	50%	100%	100%
Specificity (= TN/(TN+FP))	100%	100%	100%	100%	100%	100%	92%	100%	100%	94%	100%	78%
Accuracy (= (TP+TN)/(TP+FP+FN+TN))	100%	100%	100%	100%	100%	100%*	90%*	100%	100%	73%	100%	89%
Precision (= TP/(TP+FP)	100%	100%	100%	100%	100%	100%	94%	100%	100%	88%	100%	83%

TP, true positive cases; TN, true negative cases; FP, false positive cases; FN, false negative cases;*, not informative cases were excluded from the calculation of accuracy.

Supplementary Table 7: Detection of *IDH1* and *IDH2* mutations using duplex and multiplex ddPCR assays.

a

No.	Diagnosis	IDH1 Mutation	IDH1 Duplex MAF* [%]	IDH1 Multiplex MAF* [%]
1	Oligodendroglioma, IDH-mutant, and 1p/19q-codeleted, CNS WHO grade 2	IDH1 R132H	29.1	28.5
2	Oligodendroglioma, IDH-mutant, and 1p/19q-codeleted, CNS WHO grade 2	IDH1 R132H	23.3	23.1
3	Oligodendroglioma, IDH-mutant, and 1p/19q-codeleted, CNS WHO grade 2	IDH1 R132H	41.5	39.6
4	Oligodendroglioma, IDH-mutant, and 1p/19q-codeleted, CNS WHO grade 2	IDH1 R132H	41.1	42.7
5	Oligodendroglioma, IDH-mutant, and 1p/19q-codeleted, CNS WHO grade 2	IDH1 R132H	35.8	36.5
6	Oligodendroglioma, IDH-mutant, and 1p/19q-codeleted, CNS WHO grade 3	IDH1 R132H	36.6	35.2
7	Oligodendroglioma, IDH-mutant, and 1p/19q-codeleted, CNS WHO grade 3	IDH1 R132H	48.3	47.3
8	Oligodendroglioma, IDH-mutant, and 1p/19q-codeleted, CNS WHO grade 3	IDH1 R132H	43.9	42.9
9	Oligodendroglioma, IDH-mutant, and 1p/19q-codeleted, CNS WHO grade 3	IDH1 R132H	46.4	44.9
10	Oligodendroglioma, IDH-mutant, and 1p/19q-codeleted, CNS WHO grade 3	IDH1 R132H	19.8	19.4
11	Oligodendroglioma, IDH-mutant, and 1p/19q-codeleted, CNS WHO grade 3	IDH1 R132H	39.3	39.6
12	Astrocytoma, IDH-mutant, CNS WHO grade 4	IDH1 R132H	48.3	48.3
13	Astrocytoma, IDH-mutant, CNS WHO grade 2	IDH1 R132C	35.3	35.6
14	Astrocytoma, IDH-mutant, CNS WHO grade 2	IDH1 R132G	41.7	42.4
15	Astrocytoma, IDH-mutant, CNS WHO grade 2	IDH1 R132L	25.0	25.4
16	Astrocytoma, IDH-mutant, CNS WHO grade 4	IDH1 R132S	29.2	29.5

b

No.	Diagnosis	IDH2 Mutation	IDH2 Duplex MAF [%]	IDH2 Multiplex MAF [%]
1	Astrocytoma, IDH-mutant, CNS WHO grade 2	IDH2 R172K	5.0	5.4
2	Astrocytoma, IDH-mutant, CNS WHO grade 4	IDH2 R172K	48.2	48.4
3	Oligodendroglioma, IDH-mutant, and 1p/19q-codeleted, CNS WHO grade 3	IDH2 R172K	57.1	54.2
4	Oligodendroglioma, IDH-mutant, and 1p/19q-codeleted, CNS WHO grade 3	IDH2 R172K	41.0	42.7
5	Oligodendroglioma, IDH-mutant, and 1p/19q-codeleted, CNS WHO grade 3	IDH2 R172M	37.5	43.7

**MAF*, mutant allele frequency

Supplementary Table 8: Detection of BRAF V600E and BRAF V600K mutations using duplex ddPCR assays.

No.	Diagnosis	BRAF status determined by ddPCR	BRAF V600E mean MAF* [%]	BRAF V600K mean MAF* [%]
1	Pilocytic astrocytoma, CNS WHO grade 1	V600E	22.1	0.0
2	others (ganglioglioma, CNS WHO grade 1)	V600E	5.3	n.a.
3	others (pleomorphic xanthoastrocytoma, CNS WHO grade 2)	V600E	36.6	n.a.
4	Glioblastoma, IDH-wildtype, CNS WHO grade 4	V600E	44.8	0.1
5	Glioblastoma, IDH-wildtype, CNS WHO grade 4	V600E	21.1	0.0
6	Metastasis of a malignant melanoma	V600E	50.2	n.a.
7	Metastasis of a malignant melanoma	V600K	0.3	31.4
8	Pilocytic astrocytoma, CNS WHO grade 1	wt	0.0	n.a.
9	Pilocytic astrocytoma, CNS WHO grade 1	wt	0.0	0.0
10	Pilocytic astrocytoma, CNS WHO grade 1	wt	0.0	0.0
11	Pilocytic astrocytoma, CNS WHO grade 1	wt	0.0	n.a.
12	Astrocytoma, IDH-mutant, CNS WHO grade 2	wt	0.0	n.a.
13	Glioblastoma, IDH-wildtype, CNS WHO grade 4	wt	0.0	n.a.
14	Glioblastoma, IDH-wildtype, CNS WHO grade 4	wt	0.0	0.0
15	Glioblastoma, IDH-wildtype, CNS WHO grade 4	wt	0.0	n.a.
16	Glioblastoma, IDH-wildtype, CNS WHO grade 4	wt	0.0	n.a.
17	Glioblastoma, IDH-wildtype, CNS WHO grade 4	wt	0.0	n.a.
18	Glioblastoma, IDH-wildtype, CNS WHO grade 4	wt	0.0	n.a.
19	Glioblastoma, IDH-wildtype, CNS WHO grade 4	wt	0.0	n.a.
20	Glioblastoma, IDH-wildtype, CNS WHO grade 4	wt	0.0	n.a.
21	Glioblastoma, IDH-wildtype, CNS WHO grade 4	wt	0.0	n.a.
22	Glioblastoma, IDH-wildtype, CNS WHO grade 4	wt	0.0	n.a.

**MAF*, mutant allele frequency; *n.a.*, not analysed; *wt,* wildtype

Supplementary Table 9: Detection of H3-3A p.K28M and H3-3A p.G35R mutations using duplex ddPCR assays.

No.	Diagnosis	H3-3A status determined by ddPCR	H3-3A p.K28M (c.83A>T) MAF* [%]	H3-3A p.G35R (c.103G>A) MAF* [%]	H3-3A p.G35R c.103G>C) MAF* [%]	H3-3A p.G35V (c.104G>T) MAF* [%]
1	Diffuse midline glioma, H3 K27-altered	p.K28M (c.83A>T)	35.92	0.05	n.a.	n.a.
2	Diffuse midline glioma, H3 K27-altered	p.K28M (c.83A>T)	46.89	0.25	n.a.	n.a.
3	Diffuse midline glioma, H3 K27-altered	p.K28M (c.83A>T)	32.90	0.45	n.a.	n.a.
4	Diffuse midline glioma, H3 K27-altered	p.K28M (c.83A>T)	80.70	n.a.	n.a.	n.a.
5	Diffuse midline glioma, H3 K27-altered	p.K28M (c.83A>T)	38.80	n.a.	n.a.	n.a.
6	Diffuse midline glioma, H3 K27-altered	p.K28M (c.83A>T)	62.70	n.a.	n.a.	n.a.
7	Diffuse midline glioma, H3 K27-altered	p.K28M (c.83A>T)	41.10	n.a.	n.a.	n.a.
8	Diffuse midline glioma, H3 K27-altered	p.K28M (c.83A>T)	43.50	n.a.	n.a.	n.a.
9	Diffuse hemispheric glioma, H3 G34-mutant	p.G35R (c.103G>A)	0.02	38.00	n.a.	n.a.
10	Diffuse hemispheric glioma, H3 G34-mutant	p.G35R (c.103G>A)	0.00	36.35	n.a.	n.a.
11	Diffuse hemispheric glioma, H3 G34-mutant	p.G35R (c.103G>A)	n.a.	38.10	n.a.	0.00
12	Diffuse hemispheric glioma, H3 G34-mutant	p.G35R (c.103G>C)	n.a.	n.a.	43.70	n.a.
13	Diffuse hemispheric glioma, H3 G34-mutant	p.G35V (c.104G>T)	n.a.	n.a.	n.a.	43.80
14	Oligodendroglioma, IDH-mutant, and 1p/19q-codeleted, CNS WHO grade 3	wt	0.00	0.32	n.a.	n.a.
15	Glioblastoma, IDH-wildtype, CNS WHO grade 4	wt	0.01	0.04	n.a.	n.a.
16	Astrocytoma, IDH-mutant, CNS WHO grade 4	wt	0.00	0.06	n.a.	n.a.

**MAF*, mutant allele frequency; *n.a.*, not analysed; *wt,* wildtype

Supplementary Table 10: Comparison of the experimentally detected mean allele frequency (AF) of deleted SNP loci on 1p and 19q versus the AF calculated from the mutant allele frequency (MAF) of *TERTp* (a) or *IDH1/2* (b) mutations.

a

No.	Diagnosis	Mean MAF TERTp	TCC[a] calculated from MAF TERTp	AF 1p19q calculated* from MAF TERTp [%]	Mean AF 1p19q detected [%]	ΔAF[b] 1p19q (AF calculated-AF detected)
1	Oligodendroglioma, IDH-mutant, and 1p/19q-codeleted, CNS WHO grade 2	28.7	57.4	29.9	24.6	5.3
2	Oligodendroglioma, IDH-mutant, and 1p/19q-codeleted, CNS WHO grade 2	20.9	41.8	36.8	29.4	7.4
3	Oligodendroglioma, IDH-mutant, and 1p/19q-codeleted, CNS WHO grade 2	40.5	81.0	16.0	14.4	1.6
4	Oligodendroglioma, IDH-mutant, and 1p/19q-codeleted, CNS WHO grade 2	22.7	45.4	35.3	36.7	-1.4
5	Oligodendroglioma, IDH-mutant, and 1p/19q-codeleted, CNS WHO grade 2	34.0	68.0	24.2	20.7	3.5
6	Oligodendroglioma, IDH-mutant, and 1p/19q-codeleted, CNS WHO grade 2	39.4	78.8	17.5	14.8	2.7
7	Oligodendroglioma, IDH-mutant, and 1p/19q-codeleted, CNS WHO grade 3	40.5	81.0	16.0	15.6	0.3
8	Oligodendroglioma, IDH-mutant, and 1p/19q-codeleted, CNS WHO grade 3	46.7	93.4	6.2	5.4	0.8
9	Oligodendroglioma, IDH-mutant, and 1p/19q-codeleted, CNS WHO grade 3	42.5	85.0	13.0	16.1	-3.1
10	Oligodendroglioma, IDH-mutant, and 1p/19q-codeleted, CNS WHO grade 3	30.2	60.4	28.4	26.2	2.1
11	Oligodendroglioma, IDH-mutant, and 1p/19q-codeleted, CNS WHO grade 3	41.6	83.2	14.4	20.4	-6.0
12	Oligodendroglioma, IDH-mutant, and 1p/19q-codeleted, CNS WHO grade 3	45.3	90.6	8.6	4.3	4.3
13	Oligodendroglioma, IDH-mutant, and 1p/19q-codeleted, CNS WHO grade 3	19.0	38.0	38.3	25.9	***12.4***
14	Oligodendroglioma, IDH-mutant, and 1p/19q-codeleted, CNS WHO grade 3	39.5	79.0	17.4	16.5	0.8
15	Oligodendroglioma, IDH-mutant, and 1p/19q-codeleted, CNS WHO grade 3	44.9	89.8	9.3	11.1	-1.8
16	Oligodendroglioma, IDH-mutant, and 1p/19q-codeleted, CNS WHO grade 3	47.0	94.0	5.7	9.0	-3.3
17	Oligodendroglioma, IDH-mutant, and 1p/19q-codeleted, CNS WHO grade 3	46.4	92.8	6.7	8.9	-2.1

b

No.	Diagnosis	Mean MAF IDH1/2	TCC[a] calculated from MAF IDH1/2	AF 1p19q calculated* from MAF IDH1/2 [%]	Mean AF 1p19q detected [%]	ΔAF[b] 1p19q (AF calculated-AF detected)
1	Oligodendroglioma, IDH-mutant, and 1p/19q-codeleted, CNS WHO grade 2	27.8	55.6	30.7	24.6	6.2
2	Oligodendroglioma, IDH-mutant, and 1p/19q-codeleted, CNS WHO grade 2	27.0	54.0	31.5	31.4	0.1
3	Oligodendroglioma, IDH-mutant, and 1p/19q-codeleted, CNS WHO grade 2	28.5	57.0	30.1	29.4	0.7
4	Oligodendroglioma, IDH-mutant, and 1p/19q-codeleted, CNS WHO grade 2	42.7	85.4	12.7	14.4	-1.7
5	Oligodendroglioma, IDH-mutant, and 1p/19q-codeleted, CNS WHO grade 2	23.1	46.2	35.0	36.7	-1.7
6	Oligodendroglioma, IDH-mutant, and 1p/19q-codeleted, CNS WHO grade 2	36.5	73.0	21.3	20.7	0.5
7	Oligodendroglioma, IDH-mutant, and 1p/19q-codeleted, CNS WHO grade 2	42.5	85.0	13.0	14.8	-1.7
8	Oligodendroglioma, IDH-mutant, and 1p/19q-codeleted, CNS WHO grade 3	40.8	81.6	15.5	15.6	-0.1
9	Oligodendroglioma, IDH-mutant, and 1p/19q-codeleted, CNS WHO grade 3	45.9	91.8	7.6	7.2	0.4
10	Oligodendroglioma, IDH-mutant, and 1p/19q-codeleted, CNS WHO grade 3	47.8	95.6	4.2	5.4	-1.2
11	Oligodendroglioma, IDH-mutant, and 1p/19q-codeleted, CNS WHO grade 3	42.7	85.4	12.7	16.1	-3.4
12	Oligodendroglioma, IDH-mutant, and 1p/19q-codeleted, CNS WHO grade 3	31.1	62.2	27.4	26.2	1.2
13	Oligodendroglioma, IDH-mutant, and 1p/19q-codeleted, CNS WHO grade 3	44.4	88.8	10.1	8.1	1.9
14	Oligodendroglioma, IDH-mutant, and 1p/19q-codeleted, CNS WHO grade 3	35.2	70.4	22.8	20.4	2.5
15	Oligodendroglioma, IDH-mutant, and 1p/19q-codeleted, CNS WHO grade 3	47.3	94.6	5.1	4.3	0.9
16	Oligodendroglioma, IDH-mutant, and 1p/19q-codeleted, CNS WHO grade 3	19.4	38.8	38.0	25.9	***12.1***
17	Oligodendroglioma, IDH-mutant, and 1p/19q-codeleted, CNS WHO grade 3	39.6	79.2	17.2	16.5	0.7
18	Oligodendroglioma, IDH-mutant, and 1p/19q-codeleted, CNS WHO grade 3	42.9	85.8	12.4	11.1	1.3
19	Oligodendroglioma, IDH-mutant, and 1p/19q-codeleted, CNS WHO grade 3	44.9	89.8	9.3	9.0	0.3
20	Oligodendroglioma, IDH-mutant, and 1p/19q-codeleted, CNS WHO grade 3	44.3	88.6	10.2	8.9	1.4

[a]*TCC*, tumor cell content was calculated as follows: mean MAF *TERTp* or *IDH1/2* [%] x 2;
*, Allele frequency of the deleted alleles on 1p19q was calculated as follows: (100-TCC)/(200-TCC) x 100;
[b], ΔAF ≥ 10.0 was marked (italics and bold).

Supplementary Table 11: Comparison of the experimentally detected mean allele frequency (AF) of deleted SNP loci on chromosome 10 versus the AF calculated from the mutant allele frequency (MAF) of *TERTp* mutations.

No.	Diagnosis	Mean MAF TERTp [%]	TCC[a] calculated from MAF TERTp [%]	AF chr10 calculated* from MAF TERTp [%]	Mean AF chr10 detected [%]	Δ[b]AF chr10 (AF calculated - AF detected)
1	Glioblastoma, IDH-wildtype, CNS WHO grade 4	34.2	68.4	24.0	22.2	1.8
2	Glioblastoma, IDH-wildtype, CNS WHO grade 4	27.9	55.8	30.7	31.6	-0.9
3	Glioblastoma, IDH-wildtype, CNS WHO grade 4	29.4	58.8	29.2	27.6	1.6
4	Glioblastoma, IDH-wildtype, CNS WHO grade 4	42.4	84.8	13.2	11.4	1.8
5	Glioblastoma, IDH-wildtype, CNS WHO grade 4	42.8	85.6	12.6	13.1	-0.5
6	Glioblastoma, IDH-wildtype, CNS WHO grade 4	41.1	82.2	15.1	11.5	3.7
7	Glioblastoma, IDH-wildtype, CNS WHO grade 4	27.6	55.2	30.9	27.2	3.7
8	Glioblastoma, IDH-wildtype, CNS WHO grade 4	8.9	17.8	45.1	38.9	6.2
9	Glioblastoma, IDH-wildtype, CNS WHO grade 4	20.9	41.8	36.8	31.1	5.7
10	Glioblastoma, IDH-wildtype, CNS WHO grade 4	14.6	29.2	41.5	40.3	1.2
11	Glioblastoma, IDH-wildtype, CNS WHO grade 4	31.0	62.0	27.5	12.6	***15.0***
12	Glioblastoma, IDH-wildtype, CNS WHO grade 4	35.8	71.6	22.1	24.8	-2.6
13	Glioblastoma, IDH-wildtype, CNS WHO grade 4	37.5	75.0	20.0	22.0	-2.0

[a]*TCC*, tumor cell content was calculated as follows: mean MAF *TERTp* [%] x 2;
*, Allele frequency of the deleted alleles on chr10 was calculated as follows: (100-TCC)/(200-TCC)*100
[b], ΔAF ≥ 10.0 was marked (italics and bold).

Supplementary Table 12: Detection of the *EGFRvIII* variant using ddPCR as well as comparison of a commercially available PrimePCR™ ddPCR (a) and a self-designed (b) copy number assay for *EGFR* exon 28.

No.	Diagnosis	CN EGFR Status determined by NGS	CN EGFR exon 7 [64 bp]	CN[a] EGFR exon 28 [88 bp]	CN[b] EGFR exon 28 [64 bp]	CN EGFR Status determined by ddPCR
1	Glioblastoma, IDH-wildtype, CNS WHO grade 4	amp	65.3	27.3	49.8	amp
2	Glioblastoma, IDH-wildtype, CNS WHO grade 4	amp	27.1	26.8	29.8	amp
3	Glioblastoma, IDH-wildtype, CNS WHO grade 4	amp	71.0	100.0	111.0	vIII, amp*
4	Glioblastoma, IDH-wildtype, CNS WHO grade 4	amp	90.2	54.3	83.5	amp
5	Glioblastoma, IDH-wildtype, CNS WHO grade 4	amp	10.5	13.3	15.4	amp
6	Glioblastoma, IDH-wildtype, CNS WHO grade 4	amp	73.3	31.6	47.3	amp
7	Glioblastoma, IDH-wildtype, CNS WHO grade 4	vIII, amp	4.5	21.0	21.9	vIII, amp
8	Glioblastoma, IDH-wildtype, CNS WHO grade 4	vIII, amp	40.9	47.4	61.6	vIII, amp
9	Glioblastoma, IDH-wildtype, CNS WHO grade 4	vIII, amp	20.3	32.5	37.0	vIII, amp
10	Glioblastoma, IDH-wildtype, CNS WHO grade 4	vIII, amp	367.0	162.0	274.0	amp*
11	Glioblastoma, IDH-wildtype, CNS WHO grade 4	vIII, amp	6.2	77.5	99.8	vIII, amp
12	Glioblastoma, IDH-wildtype, CNS WHO grade 4	vIII, amp	22.3	28.4	27.8	amp*
13	Glioblastoma, IDH-wildtype, CNS WHO grade 4	vIII, amp	3.4	50.1	62.6	vIII, amp

Number 6 and 7, 8 and 9 as well as 12 and 13 are primary and recurrent tumor pairs.
CN, copy number; *NGS*, next-generation-sequencing; *amp*, EGFR amplification (copy number *EGFR* exon 28 ≥ 5.0); *vIII*, *EGFRvIII* variant; *bp*, base pair; *, discrepancy between *EGFR* status determined by NGS and ddPCR.

Supplementary Table 13: Determination of *CDKN2A* copy number in 66 glioma samples using a PrimePCR™ ddPCR *CDKN2A* copy number assay.

No.	Diagnosis	CN CDKN2A determined by ddPCR	CN CDKN2A determined by NGS or qPCR	IDH1/2 MAF [%]	TERTp MAF [%
1	Astrocytoma, IDH-mutant, CNS WHO grade 4			48.2	48.4
2	Astrocytoma, IDH-mutant, CNS WHO grade 4			47.1	wt
3	Astrocytoma, IDH-mutant, CNS WHO grade 4			n.a.	wt
4	Astrocytoma, IDH-mutant, CNS WHO grade 4			29.3	wt
5	Glioblastoma, IDH-wildtype, CNS WHO grade 4			wt	42.5
6	Glioblastoma, IDH-wildtype, CNS WHO grade 4			wt	45.5
7	Glioblastoma, IDH-wildtype, CNS WHO grade 4			wt	n.a.
8	Glioblastoma, IDH-wildtype, CNS WHO grade 4			wt	40.7
9	Glioblastoma, IDH-wildtype, CNS WHO grade 4			wt	42.8
10	Glioblastoma, IDH-wildtype, CNS WHO grade 4			wt	38.1
11	Glioblastoma, IDH-wildtype, CNS WHO grade 4			wt	34.3
12	Glioblastoma, IDH-wildtype, CNS WHO grade 4			wt	38.9
13	Glioblastoma, IDH-wildtype, CNS WHO grade 4			wt	37.0
14	Astrocytoma, IDH-mutant, CNS WHO grade 2			39.0	wt
15	Astrocytoma, IDH-mutant, CNS WHO grade 3			n.a.	n.a.
16	Glioblastoma, IDH-wildtype, CNS WHO grade 4			wt	38.5
17	Glioblastoma, IDH-wildtype, CNS WHO grade 4			wt	37.5
18	Glioblastoma, IDH-wildtype, CNS WHO grade 4			wt	30.4
19	Glioblastoma, IDH-wildtype, CNS WHO grade 4			wt	35.8
20	Glioblastoma, IDH-wildtype, CNS WHO grade 4			wt	37.7
21	Glioblastoma, IDH-wildtype, CNS WHO grade 4			wt	n.a.
22	Glioblastoma, IDH-wildtype, CNS WHO grade 4			wt	32.3
23	Glioblastoma, IDH-wildtype, CNS WHO grade 4			wt	41.4
24	Glioblastoma, IDH-wildtype, CNS WHO grade 4			wt	29.4
25	Glioblastoma, IDH-wildtype, CNS WHO grade 4			wt	27.9
26	Glioblastoma, IDH-wildtype, CNS WHO grade 4			wt	26.9
27	Glioblastoma, IDH-wildtype, CNS WHO grade 4			wt	37.0
28	Glioblastoma, IDH-wildtype, CNS WHO grade 4			wt	29.1
29	Glioblastoma, IDH-wildtype, CNS WHO grade 4			wt	39.3
30	Glioblastoma, IDH-wildtype, CNS WHO grade 4			wt	29.1
31	Glioblastoma, IDH-wildtype, CNS WHO grade 4			wt	19.0
32	Glioblastoma, IDH-wildtype, CNS WHO grade 4			wt	n.a.
33	Glioblastoma, IDH-wildtype, CNS WHO grade 4			wt	n.a.
34	Glioblastoma, IDH-wildtype, CNS WHO grade 4			wt	n.a.
35	Glioblastoma, IDH-wildtype, CNS WHO grade 4			wt	wt
36	Glioblastoma, IDH-wildtype, CNS WHO grade 4			wt	35.5
37	Glioblastoma, IDH-wildtype, CNS WHO grade 4			wt	29.1
38	Glioblastoma, IDH-wildtype, CNS WHO grade 4			wt	31.1
39	Glioblastoma, IDH-wildtype, CNS WHO grade 4			wt	37.5
40	Oligodendroglioma, IDH-mutant, and 1p/19q-codeleted, CNS WHO grade 3			40.9	40.3
41	Oligodendroglioma, IDH-mutant, and 1p/19q-codeleted, CNS WHO grade 3			34.3	29.1
42	Astrocytoma, IDH-mutant, CNS WHO grade 2			n.a.	n.a.
43	Astrocytoma, IDH-mutant, CNS WHO grade 2			37.1	wt
44	Astrocytoma, IDH-mutant, CNS WHO grade 2			43.6	wt
45	Astrocytoma, IDH-mutant, CNS WHO grade 2			30.7	wt
46	Astrocytoma, IDH-mutant, CNS WHO grade 2			35.3	wt
47	Astrocytoma, IDH-mutant, CNS WHO grade 3			38.6	wt
48	Astrocytoma, IDH-mutant, CNS WHO grade 3			34.6	wt
49	Astrocytoma, IDH-mutant, CNS WHO grade 4			40.4	wt
50	Glioblastoma, IDH-wildtype, CNS WHO grade 4			wt	20.4
51	Glioblastoma, IDH-wildtype, CNS WHO grade 4			wt	n.a.
52	Glioblastoma, IDH-wildtype, CNS WHO grade 4			wt	n.a.
53	Glioblastoma, IDH-wildtype, CNS WHO grade 4			wt	wt
54	Glioblastoma, IDH-wildtype, CNS WHO grade 4			wt	wt
55	Glioblastoma, IDH-wildtype, CNS WHO grade 4			wt	8.4
56	Glioblastoma, IDH-wildtype, CNS WHO grade 4			wt	25.4
57	Glioblastoma, IDH-wildtype, CNS WHO grade 4			wt	14.0
58	Glioblastoma, IDH-wildtype, CNS WHO grade 4			wt	38.1
59	Oligodendroglioma, IDH-mutant, and 1p/19q-codeleted, CNS WHO grade 2			32.9	30.0
60	Oligodendroglioma, IDH-mutant, and 1p/19q-codeleted, CNS WHO grade 3			45.4	44.0
61	Oligodendroglioma, IDH-mutant, and 1p/19q-codeleted, CNS WHO grade 3			32.7	29.4
62	Oligodendroglioma, IDH-mutant, and 1p/19q-codeleted, CNS WHO grade 3			34.9	35.4
63	Pilocytic astrocytoma, CNS WHO grade 1			wt	wt
64	Pilocytic astrocytoma, CNS WHO grade 1			wt	wt
65	Pilocytic astrocytoma, CNS WHO grade 1			wt	wt
66	Pilocytic astrocytoma, CNS WHO grade 1			wt	wt

CN, copy number; *NGS*, next-generation-sequencing; *MAF*, mutant allele frequency; *wt*, wildtype; *n.a.*, not analysed; *dark grey rectangle*, *CDKN2A* homozygous deletion (CN < 0.5); *light grey rectangle*, *CDKN2A* hemizygous deletion (CN ≥ 0.5 - < 1.5); *white rectangle*, no *CDKN2A* deletion (CN ≥ 1.5).

Supplementary Figures

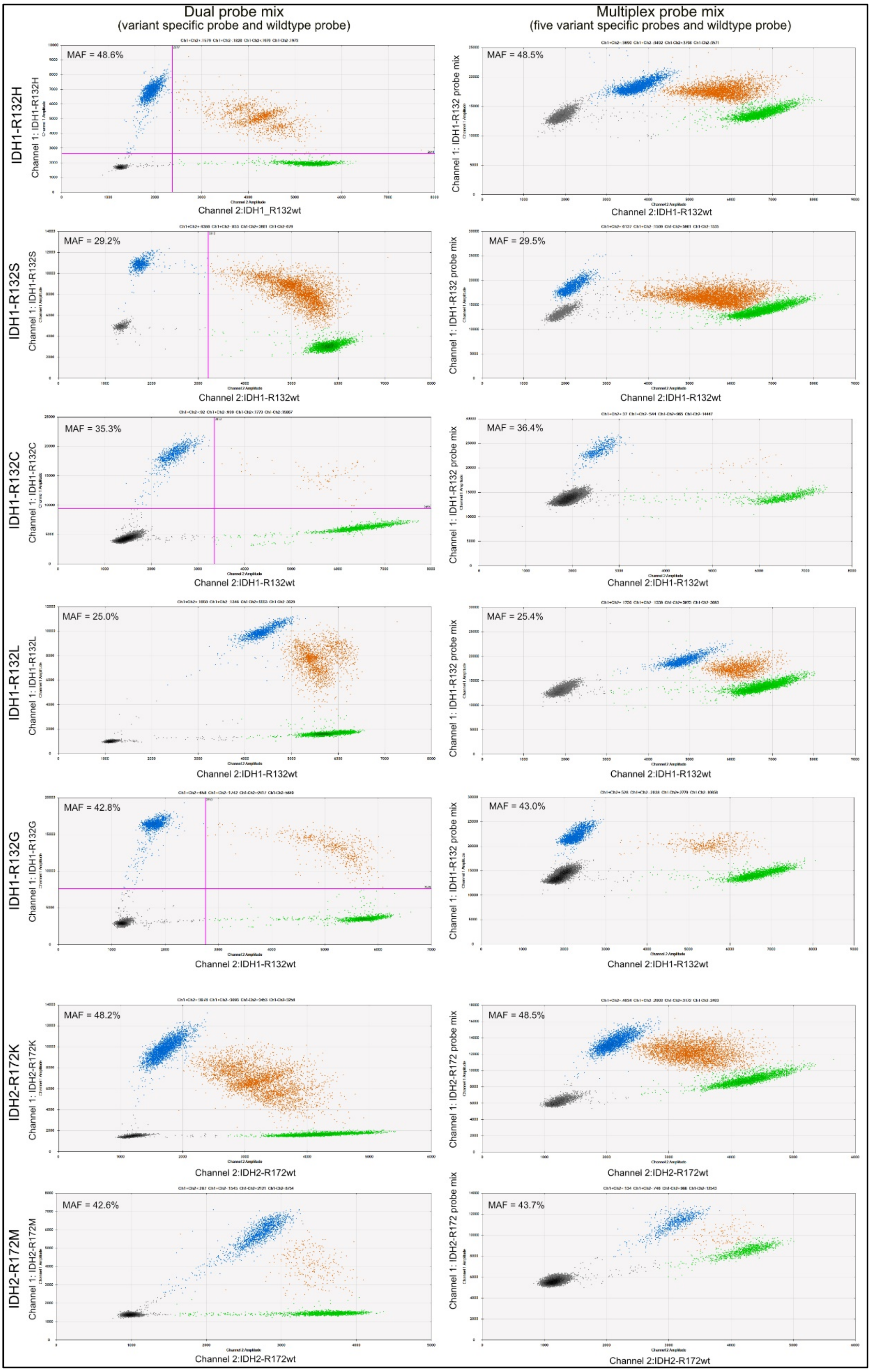

Supplementary Fig. 1 Detection of *IDH1* and *IDH2* hotspot mutations in FFPE DNA using ddPCR. The different *IDH1* and *IDH2* mutations were either detected by (*left column*) dual probe ddPCR assays of a mutation-specific and a wildtype sequence probe or by (*right column*) a mix of five *IDH1* (R132H, R132C, R132G, R132S, R132L) or *IDH2* (R172K, R172M, R172W, R172S, R172G) mutation-specific hydrolysis probes in combination with probes binding to the wildtype hotspot sequence of *IDH1* (IDH1-R132wt) or *IDH2* (IDH2-R172wt). Shown are the two-dimensional plots generated by the Quantasoft™ Software (Bio-Rad Laboratories). *X axis,* fluorescence intensity detected in the HEX-channel; *Y axis*, fluorescence intensity detected in the FAM-channel; *pink line*, threshold; *grey dots*, droplets with background fluorescence; *green dots*, droplets with fluorescence detected in the HEX-channel; *blue dots*, droplets with fluorescence detected in the FAM-channel; *orange dots*, droplets with signals in both channels.

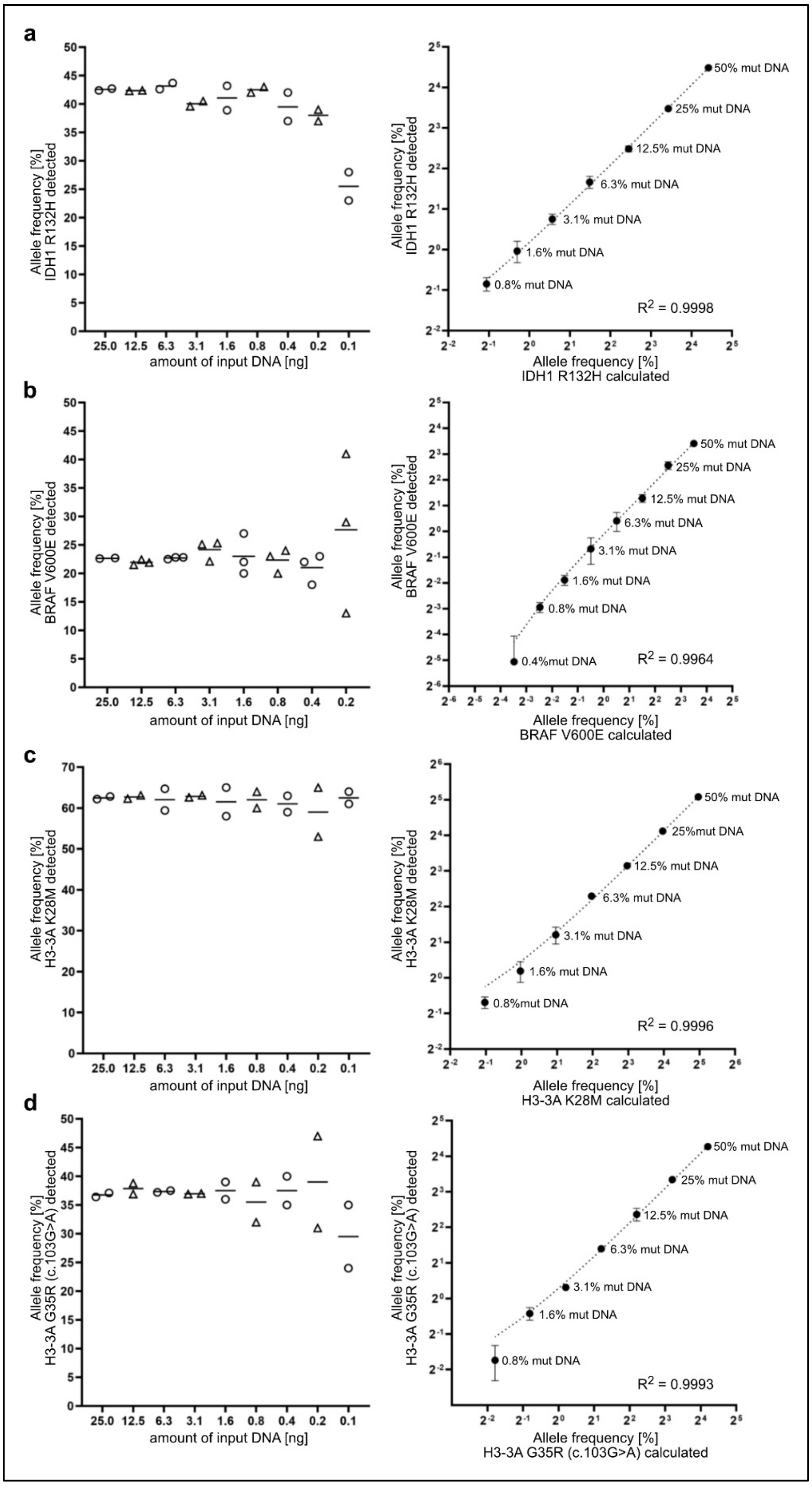

Supplementary Fig. 2 Sensitivity of ddPCR to detect IDH1 R132H (**a**), BRAF V600E (**b**) as well as H3-3A p.K28M (c.83A>T) (**c**) and H3-3A p.G35R (c.103G>A) (**d**) mutations in FFPE DNA. *Left column*, mutant allele frequency (MAF) was measured by ddPCR using different amounts of input FFPE DNA generated by serial dilution of (**a)** IDH1 R132H mutant DNA (mean MAF 42.6%), (**b**) BRAF V600E mutant DNA (mean MAF 22.7%), (**c**) H3-3A p.K28M (c.83A>T) (mean MAF 62.5%) and (**d**) H3-3A p.G35R (c.103G>A) (mean MAF 36.8%) mutant DNAs with water. *Right column*, the mutant FFPE DNAs were mixed with wildtype DNA resulting in predefined DNA samples of 50%, 25%, 12.5%, 6.3%, 3.1%, 1.6%, and 0.8% mutant (mut) DNA in a wildtype background (0.4% dilution was only measured in the case of BRAF V600E). 25 ng of total input DNA was used.

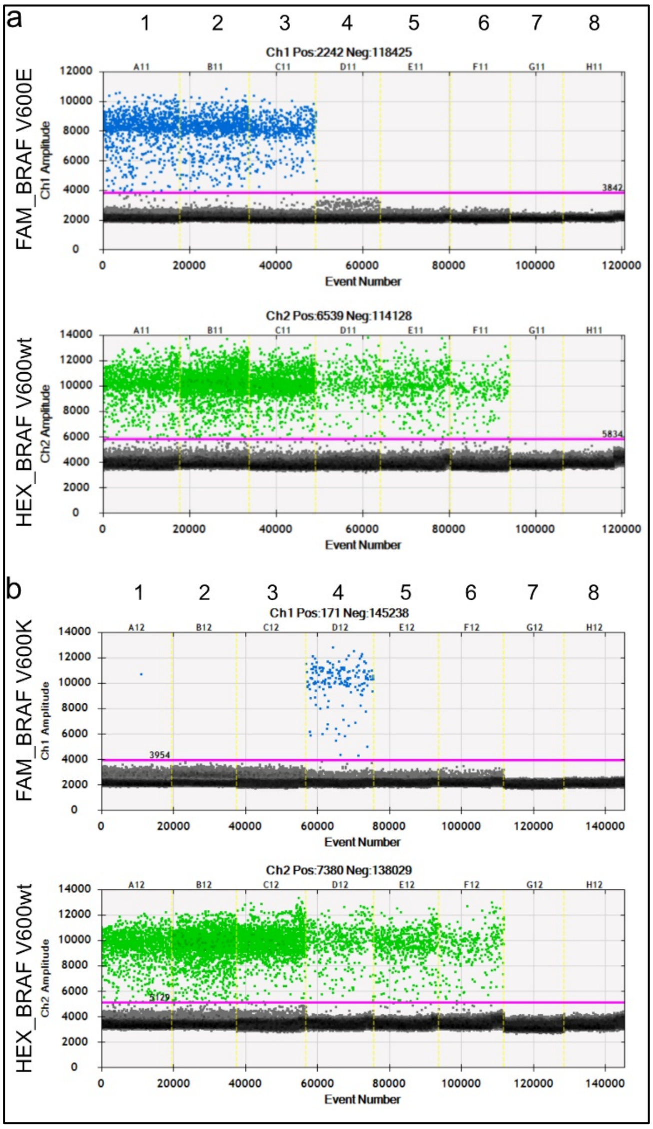

Supplementary Fig. 3 Detection of BRAF V600E (**a**) and BRAF V600K (**b**) mutations in FFPE DNA using ddPCR. The BRAF V600E and BRAF V600K mutations were detected by dual probe ddPCR assays with mutant and wildtype sequence-specific probes (for primer sequences see Supplementary Table 2). The fluorescent intensity of the droplets after amplification of a 136 bp fragment surrounding the hotspot region of *BRAF* is shown. The individual lanes correspond to: *lane 1*, BRAF V600E mutation control DNA; *lane 2 and 3*, pilocytic astrocytoma, CNS WHO grade 1, and glioblastoma, IDH-wildtype, CNS WHO grade 4, both exhibiting a BRAF V600E mutation; *lane 4*, DNA (others, metastasis of a malignant melanoma) with a BRAF V600K mutation; *lane 5 and 6*, BRAF wildtype control DNA; *lane 7, and 8*, no template control. One-dimensional plots: *X axis*, number of droplets with fluorescence; *Y axis*, fluorescence intensity detected in the FAM-channel (blue dots) and HEX-channel (green dots); *grey dots*, droplets with background fluorescence of non-incorporated probes; *pink line*, threshold. Plots were generated by the Quantasoft™ Software version 1.7.4.0917 (Bio-Rad Laboratories).

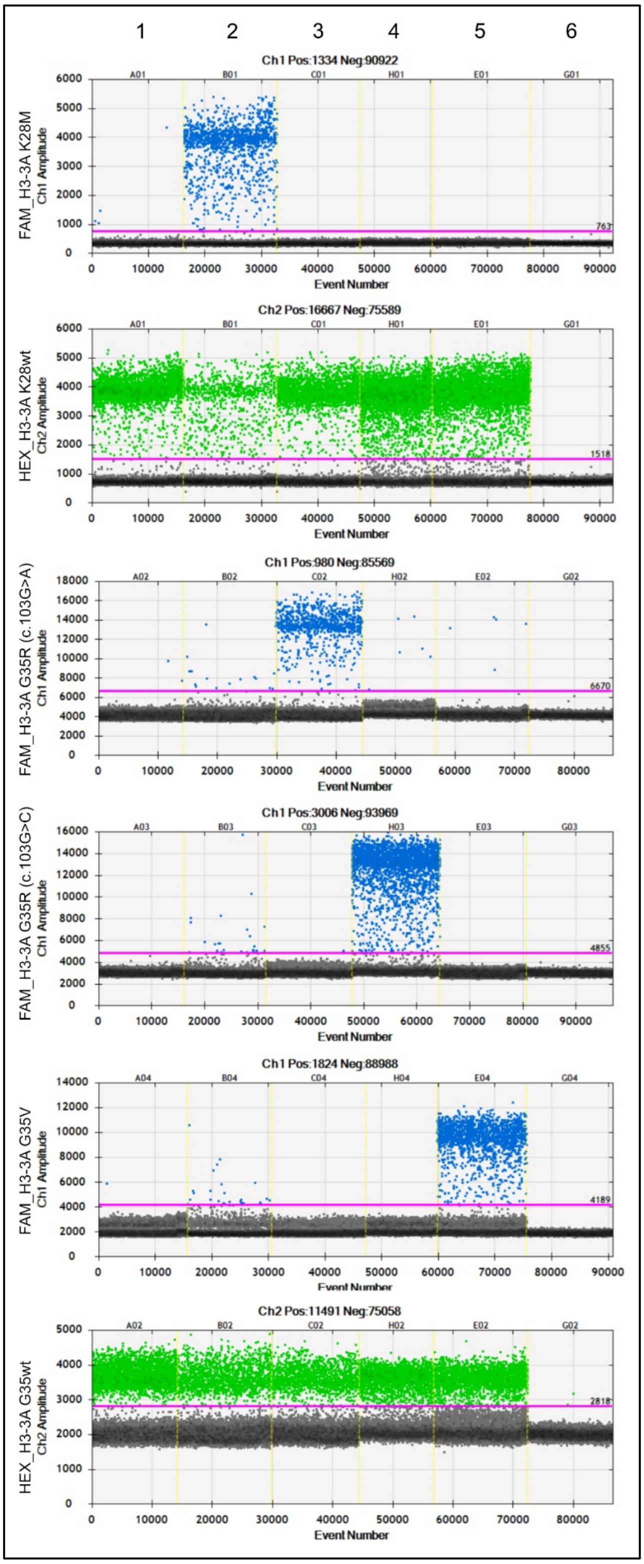

Supplementary Fig. 4 Detection of H3-3A p.K28M and H3-3A p.G35R/V mutations in FFPE DNA using ddPCR. The H3-3A p.K28M and H3-3A p.G35R and p.G35V variants were detected by dual probe ddPCR assays with mutant and wildtype sequence-specific probes (for primer sequences see Supplementary Tables 2 and 3). The individual lanes correspond to: *lane 1*, H3-3A wildtype control DNA; *lane 2*, diffuse midline glioma, H3 K27-altered with H3-3A p.K28M (c.83A>T) mutation; *lane 3-5*; diffuse hemispheric gliomas, H3 G34-mutant, with a H3-3A p. G35R (c.103G>A), H3-3A p.G35R (c.103G>C) or H3-3A p.G35V (c.104G>T) mutation, respectively; *lane 6*, no template control. One-dimensional plots: *X axis*, number of droplets with fluorescence; *Y axis*, fluorescence intensity detected in the FAM-channel (blue dots) and HEX-channel (green dots); *grey dots*, dots with background fluorescence of non-incorporated probes; *pink line*, threshold. Plots were generated by the Quantasoft™ Software version 1.7.4.0917 (Bio-Rad Laboratories).

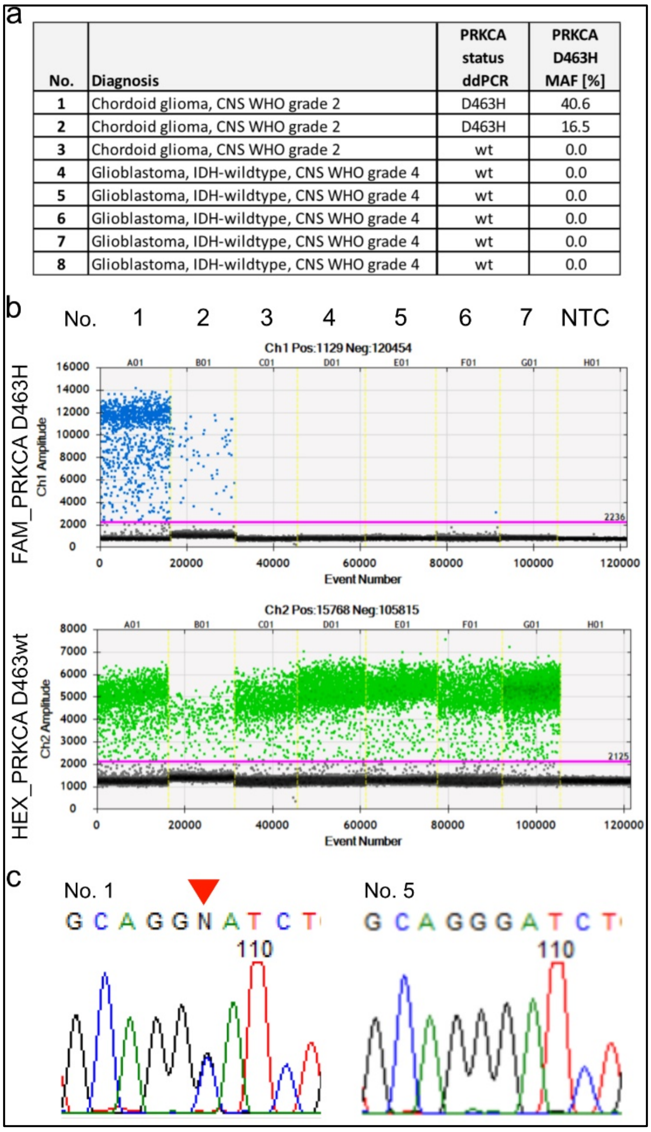

No.	Diagnosis	PRKCA status ddPCR	PRKCA D463H MAF [%]
1	Chordoid glioma, CNS WHO grade 2	D463H	40.6
2	Chordoid glioma, CNS WHO grade 2	D463H	16.5
3	Chordoid glioma, CNS WHO grade 2	wt	0.0
4	Glioblastoma, IDH-wildtype, CNS WHO grade 4	wt	0.0
5	Glioblastoma, IDH-wildtype, CNS WHO grade 4	wt	0.0
6	Glioblastoma, IDH-wildtype, CNS WHO grade 4	wt	0.0
7	Glioblastoma, IDH-wildtype, CNS WHO grade 4	wt	0.0
8	Glioblastoma, IDH-wildtype, CNS WHO grade 4	wt	0.0

Supplementary Fig. 5 Detection of the PRKCA D463H mutation in chordoid glioma FFPE tissue samples using ddPCR. **a** Results obtained in eight investigated glioma samples using a duplex ddPCR assay. **b** The PRKCA D463H variant was detected by a dual probe assay with mutant and wildtype sequence-specific probes (for primer sequences see Supplementary Table 2). Numbering of the lanes corresponds to the numbers in (**a**). The fluorescent intensity of the droplets after amplification of an 83 bp fragment surrounding the *PRKCA* hotspot region is shown. Blue droplets are only visible in the first two lanes, where the FFPE DNAs of the two chordoid glioma samples (No. 1 and 2) with PRKCA D463H mutation were analysed. One-dimensional plots: *X axis*, number of droplets with fluorescence; *Y axis*, fluorescence intensity detected in the FAM-channel (blue dots) and HEX-channel (green dots); *grey dots*, droplets with background fluorescence of non-incorporated probes; *pink line*, threshold. Plots were generated by the Quantasoft™ Software version 1.7.4.0917. *NTC*, no template control. **c** Validation of a PRKCA D463H variant using Sanger sequencing. Red arrow head pointing to the G>C exchange in the FFPE DNA of the chordoid glioma No. 1, whereas the DNA of the glioblastoma No. 5 showed the wildtype sequence at *PRKCA* codon 463.

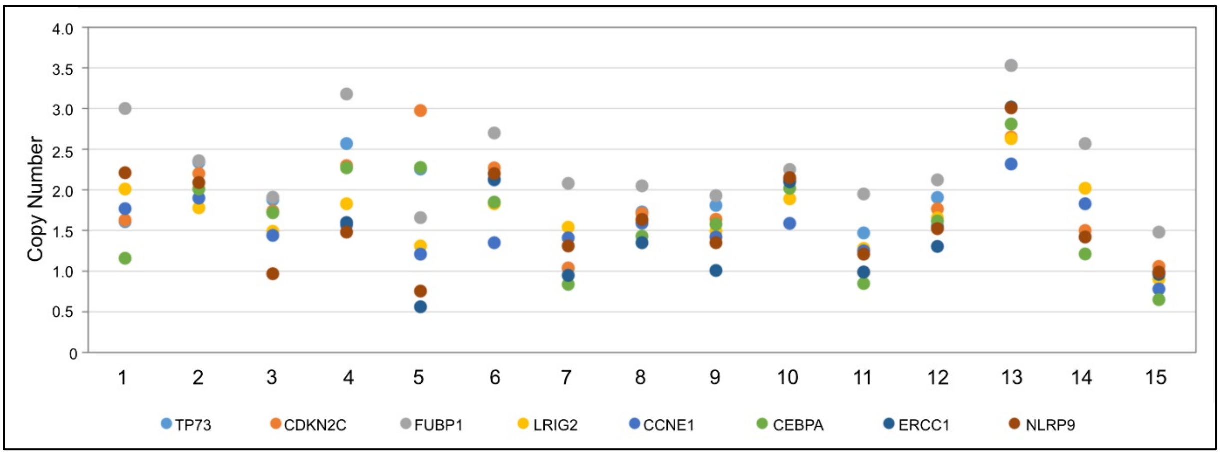

Supplementary Fig. 6 Analysis of copy number variations on chromosomal arms 1p and 19q in 15 glioma samples using ddPCR**.** For copy number analysis, four genes (*TP73*, *CDKN2C*, *FUBP1*, and *LRIG2*) on chromosomal arm 1p and four genes (*CCNE1*, *CEBPA*, *ERCC1*, and *NLRP9*) on chromosomal arm 19q were investigated in combination with an assay detecting a reference locus (*NCKAP5*, 2q21.2) (for primer sequences see Supplementary Table 2). The eight investigated genes were chosen according to Lysiak *et al.* [4]. The investigated 15 FFPE tumor DNA samples were previously validated to retain both chromosomal arms. Note that in some samples (1, 4, and 5) the copy numbers of the eight investigated loci exhibited a substantial deviation.

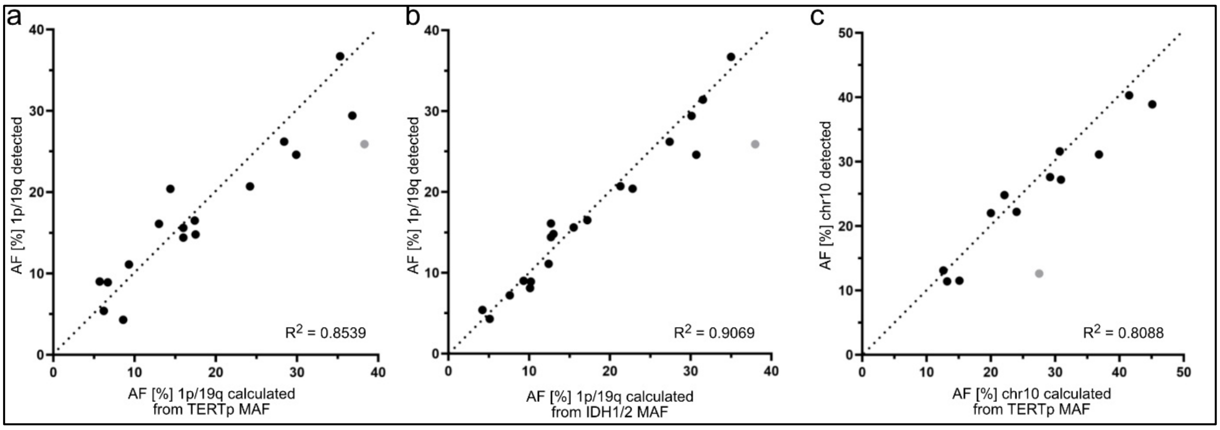

Supplementary Fig. 7 Loss of heterozygosity (LOH) on chromosomal arms 1p and 19q (a, b) as well as on chromosome 10 (c) detected by ddPCR-based SNP analysis. Pearson´s correlation analysis of the experimentally detected allele frequency (AF, frequency of the lost allele) compared to the calculated AF dependent on the tumor cell content determined by *TERTp* (a, c) or *IDH1/2* (b) mutant allele frequency (MAF) in the investigated samples. *Grey dots*, difference between AF calculated and AF detected > 10%. Note that in most cases, the expected AF values agree very well with the detected AF values.

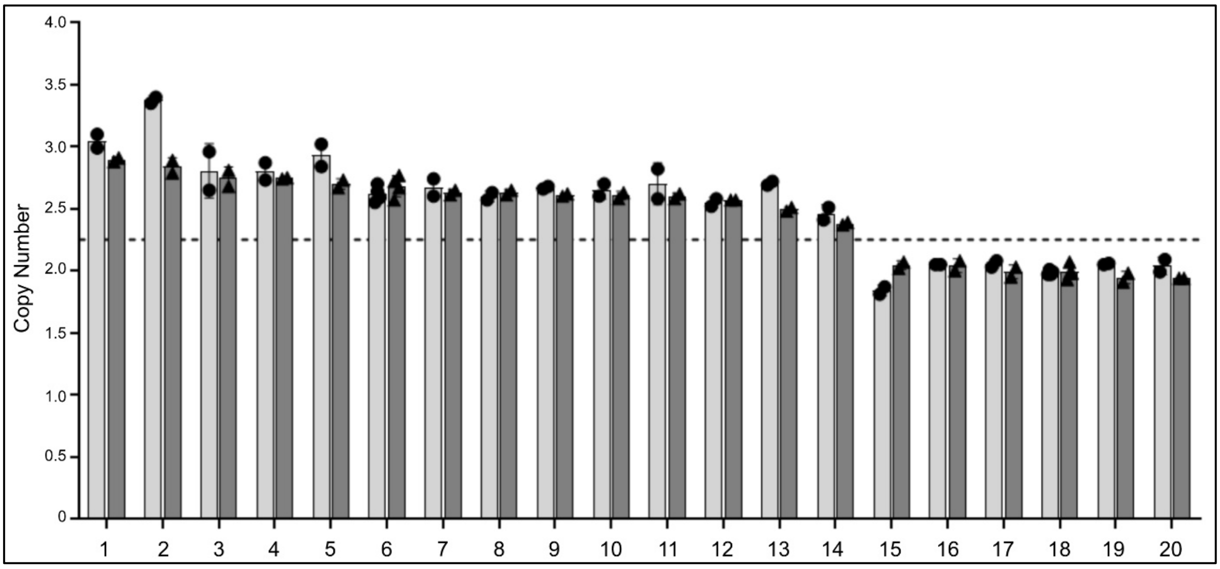

Supplementary Fig. 8 Detection of *BRAF* duplication in 20 pilocytic astrocytomas by ddPCR analysis of *UBN2* and *BRAF* copy number using the assay reported by Appay *et al.* [1]. A copy number >2.25 was considered as indicating *BRAF* gene duplication as a surrogated marker for *KIAA1549-BRAF* fusion. *Light grey rectangles*, mean copy number of *UBN2*; *dark grey rectangles*, mean copy number of *BRAF*; *dashed line*, threshold at a copy number of 2.25. All results were independently validated by other methods (see text).

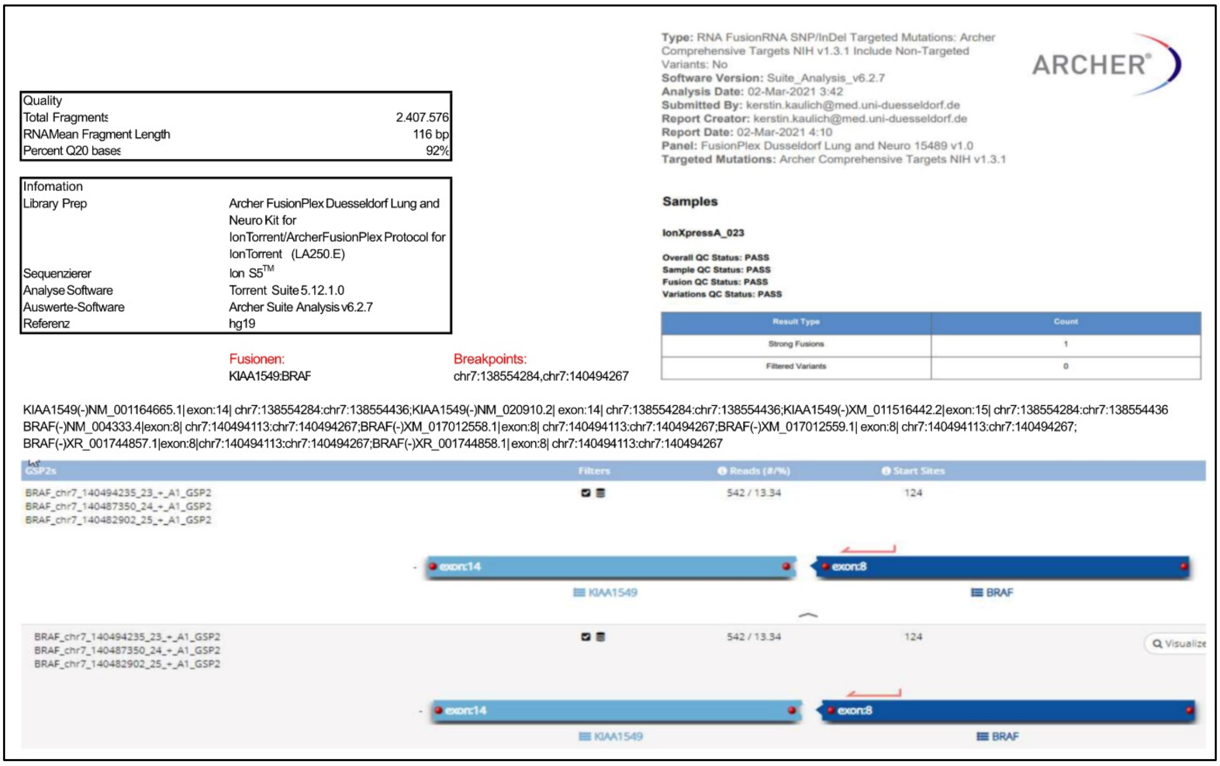

Supplementary Fig. 9 Job Report file of case 1 using the ARCHER® FusionPlex Panel and Archer Analysis software v6.2.7. The pilocytic astrocytoma (case 1 in Suppl. Fig. 7) exhibited a fusion of *KIAA1549* exon 14 and *BRAF* exon 8 detected by ddPCR but not by reverse transcriptase-PCR with commonly used primer pairs described before [5].

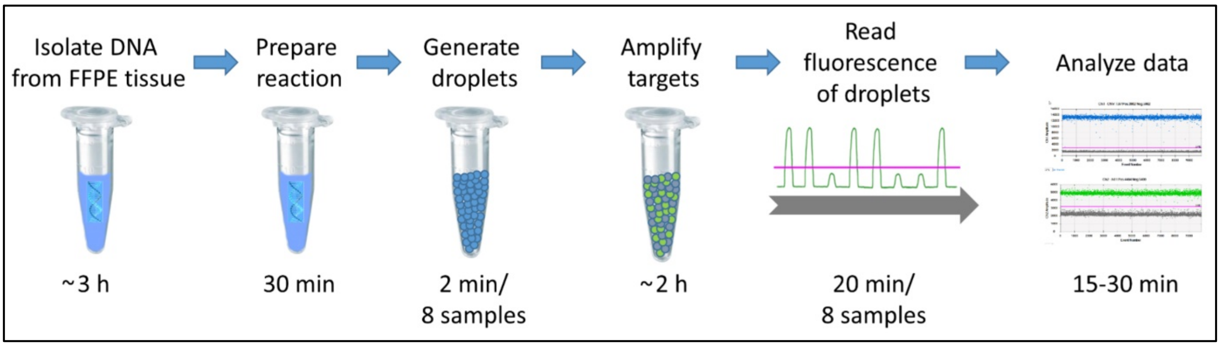

Supplementary Fig. 10 The general workflow for the ddPCR-based molecular diagnostics.

References

1. Appay R, Fina F, Macagno N et al. (2018) Duplications of KIAA1549 and BRAF screening by Droplet Digital PCR from formalin-fixed paraffin-embedded DNA is an accurate alternative for KIAA1549-BRAF fusion detection in pilocytic astrocytomas. Mod Pathol. https://doi.org/10.1038/s41379-018-0050-6
2. Hindson BJ, Ness KD, Masquelier DA et al. (2011) High-throughput droplet digital PCR system for absolute quantitation of DNA copy number. Anal Chem 83:8604–8610. https://doi.org/10.1021/ac202028g
3. Hirano M, Ohka F, Maeda S et al. (2018) A novel high-sensitivity assay to detect a small fraction of mutant IDH1 using droplet digital PCR. Brain Tumor Pathol 35:97–105. https://doi.org/10.1007/s10014-018-0310-7
4. Lysiak M, Radke K, Malmström A et al. (2017) P03.15 Detection of 1p19q co-deletion in oligodendrogliomas with droplet digital PCR. Neuro-Oncology 19:iii36. https://doi.org/10.1093/neuonc/nox036.130
5. Tian Y, Rich BE, Vena N et al. (2011) Detection of KIAA1549-BRAF fusion transcripts in formalin-fixed paraffin-embedded pediatric low-grade gliomas. J Mol Diagn 13:669–677. https://doi.org/10.1016/j.jmoldx.2011.07.002
